# Effects of Different Citrus Varieties and Harvesting Time on the Quality of Citrus Dark Tea

**DOI:** 10.3390/foods14183181

**Published:** 2025-09-12

**Authors:** Fuwei Guo, Yuanfang Jiao, De Zhang, Zhi Yu, Dejiang Ni, Han Huang, Yuqiong Chen

**Affiliations:** 1 National Key Laboratory for Germplasm Innovation and Utilization for Fruit and Vegetable Horticultural Crops, College of Horticulture & Forestry Sciences, Huazhong Agricultural University, Wuhan 430070, China; guofuwei2023@163.com (F.G.); 13598072062@163.com (Y.J.); zhangde@mail.hzau.edu.cn (D.Z.); yuzhi@mail.hzau.edu.cn (Z.Y.); nidj@mail.hzau.edu.cn (D.N.); 2 Department of Geography, University College London, London WC1E 6BT, UK

**Keywords:** green brick tea, orange dark tea, peach leaf orange, sensory quality, active ingredients, function

## Abstract

The genus *Citrus* consists of Tangerines (*Citrus × reticulata*), Mandarins (*Citrus × reticulata*), Oranges (*Citrus × sinensis*), Grapefruits (*Citrus maxima*), Lemons (*Citrus × limon*), and other citrus fruits. This study investigated the effects of 74 different citrus species and harvesting time on the quality of citrus dark tea using Hubei green brick tea as the raw material. Oranges (*Citrus × sinensis*) were found to outperform other species in improving the quality of citrus dark tea. Additionally, the impact of orange harvesting time (August to December) on the quality of orange dark tea was investigated by using the Peach leaf orange with the highest sensory score as the subject of the study. Results revealed that harvesting time can considerably impact the flavor quality of orange dark tea, but not its infusion color or scent. Specifically, the orange dark tea processed with September-harvested Peach leaf orange exhibited a strong fruity scent, a sweet and smooth flavor, and a harmonious tea and fruit aroma. With the delay of harvesting time, a downtrend was observed in the content of soluble proteins, flavonoids, hesperidin, synephrine, and limonin, as well as total volatile components, with the largest decrease in synephrine and flavonoids. However, the polysaccharide content increased in the peel of Peach leaf orange. Functional analysis revealed that the delay of harvesting time can diminish the inhibitory capacity of orange-dark tea on α-glucosidase and α-amylase. Organoleptic quality and physiological activity analysis demonstrated September as the optimal time for harvesting Peach leaf oranges for processing orange dark tea.

## 1. Introduction

Citrus belongs to the genus *Citrus* in the family Rutaceae, and its varieties primarily include Tangerines (*Citrus × reticulata*), Mandarins (*Citrus × reticulata*), Oranges (*Citrus × sinensis*), Grapefruits (*Citrus maxima*), Lemons (*Citrus × limon*), and others [1]. Citrus peels are rich in bioactive compounds such as flavonoids, essential oils, and polysaccharides, with flavonoids being the most abundant. Flavonoids mainly include flavones, flavanones, flavanols, flavonols, isoflavones, polymethoxyflavones, anthocyanins, hesperidin, naringin, and nobiletins [2]. The flavonoids in citrus peel have been confirmed to possess various beneficial effects, including antioxidant, anti-obesity, anti-atherosclerotic, anti-inflammatory, and anti-cancer properties [3,4]. Additionally, the essential oil components in citrus peel are rich in terpene hydrocarbons, esters, aldehydes, ketones, alcohols, and volatile organic acids, which exhibit multiple biological activities such as antibacterial, antidepressant, and insecticidal/anthelmintic effects [5]. Dark tea, one of the six major tea categories in China, belongs to the post-fermented tea type. It is abundant in active compounds like polysaccharides and phenolic substances, and has demonstrated significant efficacy in regulating gastrointestinal function and promoting weight loss and lipid reduction [6]. The production of citrus tea has a long history in China. As early as the Tang Dynasty, Lu Yu, the “Tea Sage,” documented in *The Classic of Tea* a brewing method that involved boiling tea together with citrus peel, a practice that has continued to this day [7]. Currently, the market offers various types of citrus tea, such as lime tea, Hunan mandarin tea, green mandarin white tea, and green mandarin pu-erh. In modern citrus tea production, the fresh fruit is first perforated, and the pulp is completely removed. The empty peel is then cleaned, air-dried or oven-dried, filled with tea leaves, and subsequently undergoes de-enzyming and drying processes [8]. The combination of citrus peel and dark tea has been proven to further accelerate the fermentation of dark tea and enhance its flavor [9].

Due to the significant differences in the composition of peels among different citrus varieties, they have a substantial impact on the quality of citrus tea. A survey by Wan Lixiu et al. [10] found that the total flavonoid content in the peels of different citrus varieties varied by nearly twofold, resulting in considerable differences in their DPPH free radical scavenging effects. Zhang et al. [11] reported that the flavonoids in Mandarin Oranges (*Citrus × sinensis*) are primarily hesperidin and nobiletin, while those in Sweet Oranges (*Citrus × sinensis*) are mainly hesperidin and narirutin. Chen et al. [12] analyzed the phenolic composition and antioxidant capacity of peels from 52 citrus varieties and found significant differences among them. Additionally, the accumulation of bioactive compounds in citrus peels is influenced by fruit maturity. Some studies have shown that as maturity increases, flavonoid content in the peel decreases, while sugar content rises [13]. Other research has revealed that the total content of 17 hydrolyzed amino acids in the peel is generally higher during the young fruit and semi-mature stages than at full maturity, thereby affecting taste and flavor [14]. Aromatic compounds in the peel are also subject to seasonal variations. For example, studies have demonstrated seasonal trends in compounds such as neryl acetate, geranyl acetate, and citronellal in lemon peel [15]. However, there is limited research on the impact of specific citrus varieties and maturity stages on the quality of citrus tea production.

This study aimed to fill this research gap by using Hubei green brick tea as the raw material of dark tea and maximizing the number of citrus varieties available to select suitable citrus peels for processing citrus dark tea. Additionally, the Peach leaf orange with the highest sensory scores was used to examine the effects of orange peels with different maturity on the quality of citrus dark tea. This study provides useful information for the development of citrus dark tea.

## 2. Materials and Methods

### 2.1. Materials and Reagents

A total of 74 citrus varieties (Table S1) were obtained from the Citrus Seed Breeding Base in Zigui County, Hubei, China, and the Citrus Resource Nursery of Huazhong Agricultural University in Wuhan, China. During sampling, three trees were randomly selected from each variety, and five fruits of approximately consistent size were randomly harvested from each tree. After harvesting, the fruits were mixed thoroughly, and 10 fruits were randomly drawn to process into orange tea. Peach Leaf Oranges at different maturity stages were harvested on 20 August, 19 September, 19 October, 16 November, and 19 December 2019, from the Ban Shan Hong Planting Cooperative in Longmaxi Village, Quyuan Town, Zigui County. 10 trees from the same orchard were first selected as experimental subjects. During each sampling, five fruits of approximately consistent size were randomly harvested from each tree. After harvesting, the fruits were mixed thoroughly, and 20 fruits were randomly drawn to process into orange tea. Qingzhuan tea (a type of dark tea) was provided by Zigui Yihong Tea Co., Ltd. (Zigui, Hubei, China). Rutin, synephrine, limonin, cyclohexanone, hesperidin, bovine serum albumin (BSA), α-glucosidase, and α-glucoside were purchased from Shanghai Yuanye Bio-Technology Co., Ltd. (Shanghai, China). The DPPH, FRAP, and ABTS antioxidant kits were purchased from Suzhou Keming Biotechnology Co., Ltd. (Suzhou, Jiangsu, China). The α-amylase activity assay kit was obtained from Solarbio Co., Ltd. (Beijing, China), the phosphoric and oxalic acids were purchased from Shanghai Aladdin Biochemical Technology Co., Ltd. (Shanghai, China) and the remaining reagents were purchased from China Pharmaceutical (Group) Shanghai Chemical Reagent Company (Shanghai, China).

### 2.2. Processing of Citrus Dark Tea

**Figure 1:**
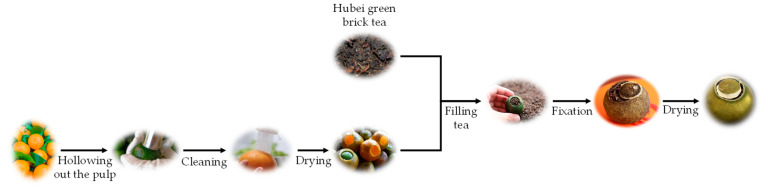
Processing flowchart of citrus dark tea.

Following a previous method with necessary adjustments [16], the citrus dark tea samples were prepared (Figure 1). Briefly, after harvesting, the fresh fruit was cleaned, followed by pulp extraction, cleaning again, and removing pulp residues. Next, the cleaned peel was dried and then filled with tea leaves to about three quarters, followed by fixation the tea samples at 50 °C for 10 min, 70 °C for 20 min, 85 °C for 10 min, 90 °C for 10 min, and 50 °C for 20 min in a 6CTH-6.0 box-type drying machine (Zhejiang Green Peak Machinery Co., Ltd., Quzhou, Zhejiang, China). After fixation, the citrus tea was spread out to cool down and then dried with a 6CTH-6.0 box-type drying machine (Zhejiang Green Peak Machinery Co., Ltd.) under the conditions of 45 °C for 2 h, 50 °C for 20 min, 45 °C for 2 h, 50 °C for 6 h, 80 °C for 10 h, 65 °C for 2 h, and 75 °C for 15 min.

### 2.3. Sensory Evaluation of Orange Dark Tea

Sensory evaluation was performed as previously reported with minor modifications [17]. First, the packaged orange tea from each treatment was opened, and the tea leaves were removed and thoroughly mixed. Simultaneously, the orange peel was crushed and uniformly blended. Then, 3 g of tea leaves and 1.2 g of crushed peel were placed in an evaluation cup, which was filled with boiling water and steeped for 5 min. The tea liquor was then strained into a tasting bowl for evaluation of its color, aroma, and taste. The aroma was primarily assessed based on sweetness intensity, fruitiness level, pleasantness, and coordination, while the taste was evaluated mainly for acidity, sweetness, bitterness, astringency, and balance (Table S2). This study was conducted in accordance with the Declaration of Helsinki and was approved by the Ethics Committee of Huazhong Agricultural University (Human Ethics application ID Number: HZAUHU-2020-0025).

### 2.4. Analysis of Major Non-Volatile Compounds in Peach Leaf Orange Dark Tea

Polyphenols in tea and orange peel [18]: After weighing and combining 0.2 g of crushed material with 10 mL of 70% methanol, the mixture was extracted in a 70 °C water bath for 10 min, followed by cooling to room temperature and centrifugation for 10 min at 3500 rpm. The supernatant was diluted to 10 mL, and the total phenolic content was analyzed using the Folin–Ciocalteu assay. The instrument used was a 722N spectrophotometer (Shanghai Jinghua Technology Instrument Co., Ltd., Shanghai, China) with the absorbance measured at 765 nm.

Free amino acids and soluble sugars in tea [18]: 0.5 g crushed sample was weighed and mixed with 50 mL of boiling water, followed by extraction in a boiling water bath for 45 min. Next, the tea infusion was filtered while hot, cooled, and brought to 100 mL. The contents of free amino acids and soluble sugars were determined using the ninhydrin assay and the anthrone-sulfuric acid colorimetric method, with absorbance measured at 570 nm and 620 nm, respectively, using a 722N spectrophotometer.

Theaflavins, thearubigins, and theabrownins in tea [17]: Ethyl acetate, NaHCO_3_, and n-butanol were used to extract the filtrate. After adding 3 g of crushed tea samples to 125 mL of boiling water, and extraction in a boiling water bath for 10 min, the samples were then filtered while still hot. A systematic assay was used to determine the amount of theaflavins, thearubigins, and theabrownins in the filtrate. Absorbance was measured at 380 nm using a 722N spectrophotometer.

Polysaccharides in orange peel [19]: After adding 0.2 g of the crushed peel to 40 mL of 80% ethanol, the mixture was reflux-extracted in a 95 °C water bath. After adding 100 mL of distilled water, extraction in a hot water bath, and filtration, the volume was then fixed at 100 mL. Finally, the anthrone-sulfuric acid method was used to identify the polysaccharides in orange peels. Absorbance was measured at 620 nm using a 722N spectrophotometer.

Flavonoid in peel [20]: 0.2 g of the ground sample was weighed and ultrasonically extracted with 40 mL of anhydrous ethanol for 30 min. After filtration, the solution was diluted to a final volume of 50 mL. The flavonoid content was determined using the aluminum nitrate method, and absorbance was measured at 380 nm with a 722N spectrophotometer.

Soluble proteins in orange peel [19]: After extracting 0.2 g of crushed sample with 100 °C boiling water for 10 min, followed by centrifugation at 3000 rpm for 10 min to collect the supernatant and fixing the volume to 50 mL. The Coomassie brilliant blue method was used to determine the soluble proteins, and absorbance was measured at 595 nm using a 722N spectrophotometer.

Hesperidin, synephrine, and limonin in peel: They were simultaneously determined using high-performance liquid chromatography (HPLC) on an Agilent 1260 system (Agilent Technologies, Santa Clara, CA, USA) [21]. Briefly, 10 mL of methanol was mixed with 0.1 g of orange peel powder, followed by sonication for 30 min, filtration, fixing the volume to 10 mL, and then filtration of 1 mL through a 0.22 μm filter membrane. HPLC conditions: mobile phase A, aqueous phosphoric acid pH = 3.7; mobile phase B, methanol: acetonitrile = 1:1; chromatographic column, Agilent ZORBAX SB-C 18 (250 mm × 4.6 mm × 5 μm); flow rate, 1 mL-min-1; column temperature, 35 °C; injection volume, 5 μL; detection wavelength, 210 nm and 283 nm; HPLC circumstances, A:B = 25:75 for 30 min, 5:95 for 30–40 min, and 95:5 for 5–10 min. Quantitative analysis was performed using the external standard method. Authentic standards of hesperidin, synephrine, and limonin were used to prepare a series of five standard solutions at varying concentrations. Following analysis by HPLC, calibration curves were constructed by plotting the peak areas of each compound against their corresponding concentrations. All calibration curves demonstrated good linearity, with correlation coefficients (R^2^) exceeding 0.99. The target compounds in the test samples were determined by substituting the measured peak areas into the respective calibration curve equations.

### 2.5. Volatile Content of Peach Leaf Orange Dark Tea

The fragrance components were extracted using headspace solid-phase microextraction (HS-SPME; Thermo Fisher Scientific, Waltham, MA, USA) [22]. Briefly, the DVB/CAR/PDMS extraction fiber was inserted into the GC inlet and pre-conditioned for 30 min at 250 °C. After a 20 mL headspace bottle was filled with 1 g of orange tea powder, 5 mL of boiling saturated NaCl solution, and 1 mL of cyclohexanone internal standard, the bottle stopper was then quickly closed. Finally, the headspace vial was—submerged in a 60 °C water bath for 1 h. Chromatography conditions: the column, a DB-5MS (30 mm × 0.25 mm × 0.22 μm) with an inlet temperature of 230 °C; the carrier gas, high purity helium with a purity of ≥99.99%; the column flow rate,1.0 mL/min; the heating process, 45 °C initially, up to 80 °C at 7 °C/min, 90 °C at 2 °C/min and hold for 2 min, 100 °C at 3 °C/min and hold for 2 min, 130 °C at 3 °C/min and hold for 2 min, 150 °C at 3 °C/min and hold for 5 min, 230 °C at 10 °C/min and hold for 5 min, and finally, 40 °C without shunt injection.

Mass spectrometric analysis was conducted under electron ionization (EI) conditions with an electron energy of 70 eV, an ion source temperature of 230 °C, and a mass scanning range of *m*/*z* 30–500; chromatographic deconvolution of co-eluting peaks was subsequently performed using AMDIS (Version 2.72) software.

Qualitative and quantitative analysis: mass spectral data corresponding to chromatographic peaks in the total ion chromatogram were extracted and matched against both the NIST standard mass spectral database and previously published mass spectral data of tea aroma compounds. Identification of volatile compounds was conducted based on retention index (RI, determined using a C_5_–C_25_ n-alkane series), retention time, and mass spectral fragment pattern similarity. Relative quantification of each component was subsequently carried out using an internal standard method to obtain their relative concentrations.

### 2.6. Antioxidant Capacity of Peach Leaf Orange Dark Tea

Three kits (ABTS, FRAP, and DPPH, Suzhou Keming Biotechnology Co., Ltd. (Suzhou, Jiangsu, China)) were used to assess the antioxidant capacity of Peach leaf orange dark tea. Briefly, each sample powder (0.1 g) was weighed into a 10 mL centrifuge tube, followed by adding 10 mL of boiling distilled water, heating in a boiling water bath for 10 min, and then centrifuging for 5 min to collect the supernatant. Finally, the assay was performed according to the manufacturer’s instructions using the provided kit [23]:

FRAP assay: The working solution was prepared according to the manufacturer’s instructions. Subsequently, 190 μL of the working solution was added to each well of a 96-well plate, followed by the addition of 10 μL of the blank control or test samples. After incubation at room temperature for 20 min, the absorbance was determined at 593 nm using a microplate reader (ELX-800, Bio-Tek Instruments Inc., Winooski, VT, USA). The inhibitory activity was calculated using Equation (1):

ΔA = A_assay_ − A_blank_ (1)

ABTS assay: The working solution was prepared according to the manufacturer’s instructions. Then, 190 μL of the working solution was aliquoted into a 96-well plate, followed by the addition of 10 μL of the blank control or test samples. After incubation at room temperature for 10 min, the absorbance was determined at 734 nm using a microplate reader. The radical scavenging activity was calculated using Equation (2):

ΔA = A_blank1_ − A_assay1_ (2)

DPPH assay: The working solution was prepared according to the manufacturer’s instructions. Then, 380 μL of the working solution was aliquoted into a 1.5 mL microcentrifuge tube, followed by the addition of 20 μL of the blank control or test samples. After incubation at room temperature in the dark for 20 min, 200 μL of the reaction mixture was transferred to a 96-well plate. The absorbance was determined at 515 nm using a microplate reader, and the radical scavenging activity was calculated using Equation (3):

ΔA = A_blank2_ − A_assay2_ (3)

### 2.7. α-Glucosidase and α-Amylase Activities

For preparation of test solution, 0.1 g of crushed sample was weighed into a 10 mL centrifuge tube, followed by adding 10 mL of boiling distilled water and placing the tube in a boiling water bath for 10 min. After centrifugation for 5 min, the supernatant was collected and then diluted to different concentration gradients for further analysis. The α-glucosidase inhibitory activity was determined using a previous method [22]: Inhibition of enzyme activity (%) = 1 − (A1 − A2)/A0 × 100. Where A0 represents the absorbance of the enzyme after reaction with the substrate; A1, the absorbance of the enzyme reaction with the substrate after adding the sample; A2, the absorbance of the sample itself.

The inhibitory activity of α-amylase was determined as follows: α-amylase activity inhibition rate (%) = (1 − (OD_inhibition − OD_background)/(OD_reaction − OD_control)) × 100. Where OD inhibition represents the absorbance of enzyme reaction with substrate after adding the sample; OD background, the absorbance after mixing sample and substrate; OD reaction, the absorbance of enzyme reaction with substrate; OD control, the absorbance of substrate. The final results are expressed as Trolox equivalents (TE) in terms of μmol TE per gram of each sample (μmol TE/g).

### 2.8. Statistical Analysis

Every experiment was conducted in triplicate, and the data results were presented as mean X ± SD (n = 3). SPSS Statistics 26 software was used to analyze the data. Fisher’s least significant difference (LSD) test was also performed for multiple comparisons, and significant difference was determined at *p <* 0.05. To facilitate interpretation of the comprehensive dataset, multiple multivariate analytical techniques were employed. Cluster analysis and heatmap visualization were performed using TBtools-II (Version 2.138), enabling unbiased classification of samples based on sensory attributes and compositional profiles. Partial least squares-discriminant analysis (PLS-DA) and permutation tests were conducted using SIMCA (version 14.1) to identify discriminatory variables responsible for group separation and to evaluate model robustness. Correlation heatmaps were generated with ChiPlot to examine relationships between phytochemical constituents and biological activities. For volatile compound analysis, AMDIS (Version 2.72) software was employed for deconvolution of co-eluting chromatographic peaks.

## 3. Results and Discussion

### 3.1. Effects of Different Citrus Species on Sensory Quality of Citrus Dark Tea

The sensory scores of infusion color, aroma, and taste, as well as the total scores of citrus dark teas made from 74 citrus species were shown in Figure S1 and Table S2. In this study, the citrus species used in the citrus dark tea can be divided into five categories: orange, grapefruit, mandarin, tangerine, and lemon. The quality of these five citrus dark teas was statistically analyzed (Table 1) and their quality was seen to vary greatly. The tea soup profile of dark-tea-based citrus blends demonstrated predominant reddish hues, exhibiting color variations from light red, red, deep red to orange-red. The infusion color scores followed the order of orange (9.09) > lemon (8.97) > mandarin (8.93) > grapefruit (8.91) > tangerine (8.90). Further significance analysis revealed highly significant differences (*p* < 0.01) in soup color for orange versus mandarin, tangerine, grapefruit and lemon categories, and significant differences (*p* < 0.05) for lemon versus grapefruit categories.

Aroma is one of the important qualities of citrus dark tea, and the overall scores of sweetness, fruity aroma, and pleasantness followed the order of lemon (23.87) > orange (22.77) > mandarin (22.47) > grapefruit (22.22) > tangerine (20.78). Except for no significant difference (*p* > 0.05) in aroma for mandarin versus orange and grapefruit or orange versus grapefruit, the differences between the other categories were highly significant (*p* < 0.01). As citrus dark tea aroma is composed of peel and tea aroma, their coordination can directly affect its aroma quality and consumer acceptance. The inter-category coordination scores were highest for lemon (8.93), followed by orange (8.33), mandarin (8.03), and grapefruit (7.98), with the lowest score observed for tangerine (7.80). This indicated that the aroma coordination was strongest between lemon and green brick tea, followed by orange, then mandarin, while grapefruit and tangerine showed weaker coordination with green brick tea. Significance analysis revealed no significant (*p* > 0.05) difference between tangerine and grapefruit in coordination score while highly significant (*p* < 0.01) differences between the other categories. The inter-category coordination scores were highest for lemon (8.93), followed by orange (8.33), mandarin (8.03), and grapefruit (7.98), with the lowest score observed for tangerine (7.80).

Taste, the most important quality of citrus dark tea, was analyzed in terms of acidity, sweetness, bitterness, astringency, and coordination. The acidity scores were in the order of tangerine (5.51) > mandarin (5.46) > orange (5.45) > grapefruit (5.26) > lemon (4.67), indicating that the overall taste of citrus dark tea was acidic, especially lemon dark tea with a stronger acidic taste. Significance analysis showed no significant (*p* > 0.05) difference in two comparisons of tangerine, orange and mandarin, while highly significant (*p* < 0.01) difference between the other categories. Sweetness is the main factor in measuring citrus dark tea, and the sweetness scores were relatively high for tangerine (6.19), mandarin (6.08), orange (6.06) and relatively low for lemon (5.67) and grapefruit (4.86), indicating a sweeter and milder flavor for tangerine, mandarin and orange dark teas. The bitterness scores were relatively high for orange (6.44) and mandarin (6.16), medium for tangerine (5.76) and lemon (5.40), and the lowest for grapefruit (4.29), and significance analysis showed highly significant (*p* < 0.01) differences among all categories. The highest astringency score was recorded for orange (6.24), followed by mandarin (5.96), tangerine (5.78), and lemon (5.67), and the lowest for grapefruit (4.25), with significant (*p* < 0.05) differences for mandarin versus tangerine, and tangerine versus lemon, and highly significant (*p <* 0.01) differences in the remaining categories. Bitterness and astringency analysis showed the least bitterness and astringency in orange dark tea, while the most bitterness and astringency in grapefruit dark tea. Harmony is a combination of different flavors in the tea infusion, and the scores followed the order of orange (6.00) > mandarin (5.67) > tangerine (5.54) > lemon (4.67) > grapefruit (4.25), and significance analysis showed highly significant (*p* < 0.01) differences between all the categories, suggesting a considerable difference in the taste of citrus dark teas processed with different citrus species. When adding the scores of each index together, the total flavor score was the highest for orange (30.19), followed by mandarin (28.98), tangerine (27.41), lemon (26.07), and grapefruit (22.56), with highly significant (*p* < 0.01) differences between all the categories.

When combining infusion color, aroma and taste, the citrus dark teas scored in the order of orange (62.05) > mandarin (60.38) > lemon (58.90) > tangerine (56.64) > grapefruit (53.70), with highly significant differences between all the categories (*p* < 0.01). This suggests that orange could be the most suitable variety for processing dark tea, followed by mandarin, while grapefruit could be the least suitable, and most lemon and tangerine species could also be unsuitable.

### 3.2. Effects of Different Citrus Cultivars on Sensory Quality of Citrus Dark Tea

**Figure 2:**
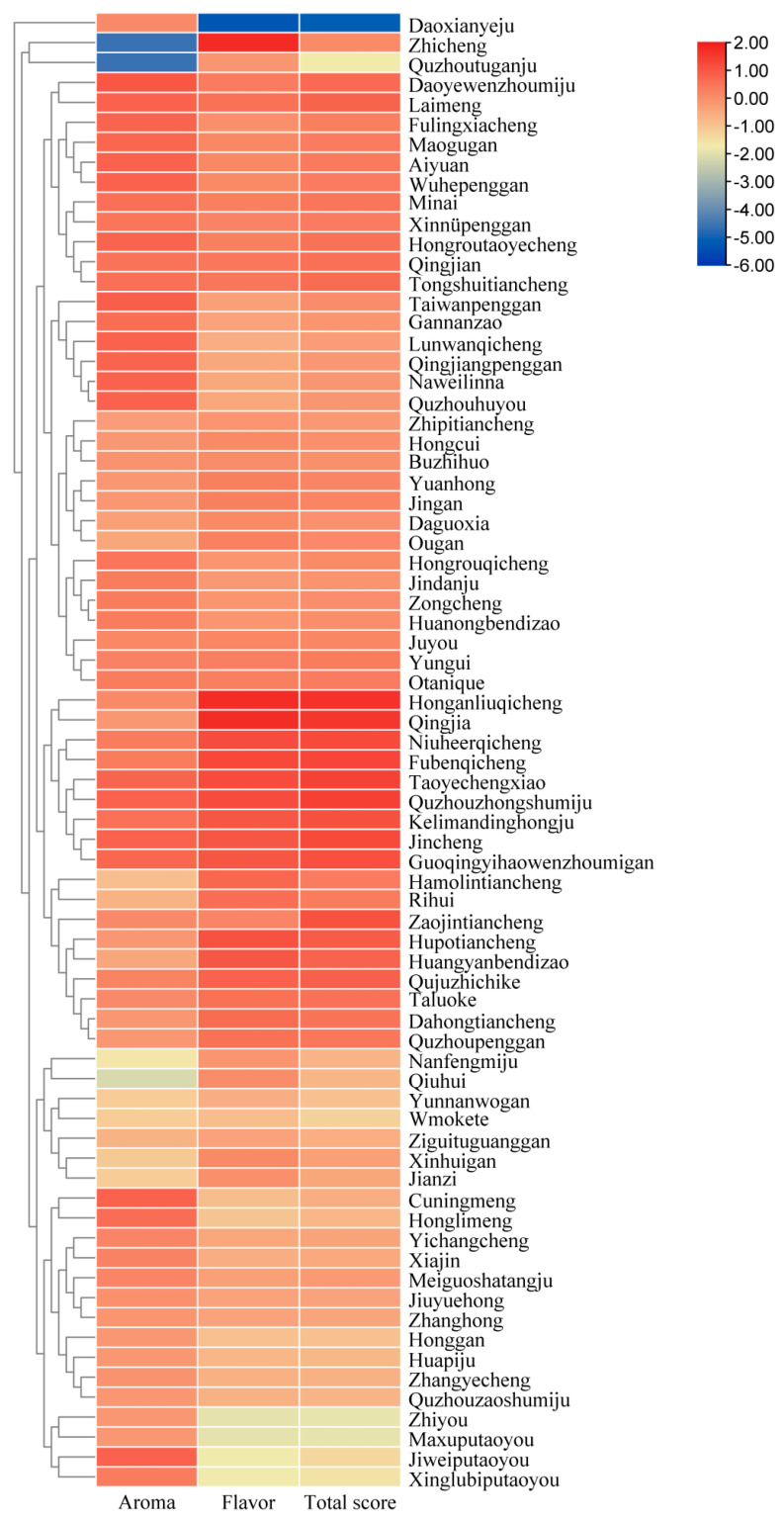
Heat map of evaluation factors of citrus dark teas processed with 74 different citrus varieties.

The aroma scores of the 74 citrus dark teas were statistically analyzed, and their average aroma score was 22.42. Further cluster analysis classified the 74 varieties into three categories based on low, medium and high scores (Figure 2), with scores below 19.32 as low, 19.32–21.32 as medium, and above 21.32 as high, accounting for 5.41%, 9.46%, and 85.14%, respectively. The clustering analysis also showed that the orange dark tea scores were in the range of 24 for the rice-leaf Wenzhou honey orange, Taiwan ponkan, Jincheng orange, Lun late navel orange, Navellina, Ehime mandarin, Seedless ponkan, Quzhou medium ripe honey orange, Lemon, Rough lemon, Cocktail grapefruit, and Quzhou hu grapefruit, indicating that these 12 cultivars are high-scoring varieties in terms of taste attributes.

The flavor scores of the 74 citrus dark teas were also statistically analyzed and their average score was 27.04. Further cluster analysis classified the 74 varieties into three categories based on low, medium and high scores (Figure 2), with <25.48 as low, 25.48–27.48 as medium, and >27.48 as high, accounting for 16.22%, 17.57%, and 66.22%, respectively. The clustering analysis showed a high score of 34 for Honganliu navel orange, Qingjia mandarin, Fuben navel orange, Peach leaf orange, Newhall navel orange, Quzhou medium ripe honey orange, Amber sweet orange, Jincheng orange, Guoqing No. 1 Wenzhou mandarin, Cremantine mandarin, and Huangyan local early mandarin, indicating that these 11 cultivars are high-scoring varieties in terms of taste attributes.

The total scores of the 74 citrus dark teas were further statistically analyzed and their average total score was 58.42. Cluster analysis classified the varieties into three categories based on low, medium and high scores (Figure 2), with scores below 56.79 as low, 56.79–58.79 as medium, and above 58.79 as high, accounting for 22.98%, 12.16%, and 64.87%, respectively. The clustering analysis showed a high score of 64 for Honganliu navel orange, Qingjia mandarin, Quzhou medium ripe honey orange, Peach leaf orange, Fuben navel orange, Jincheng orange, Newhall navel orange, Guoqing No. 1 Wenzhou mandarin, Cremantine orange, Zaojin sweet orange, Amber sweet orange, Quju mandarin, Lemon, and Huangyan local early mandarin, indicating that these 14 varieties are high scorers in overall performance. Among the high-scoring varieties, Peach leaf orange dark tea showed outstanding performance in comprehensive scoring, with good integration of tea and fruit aroma in terms of aroma, smooth and well-coordinated taste, and good coordination of orange peel and tea aroma. The fruit of Peach leaf orange, a local variety bred in Zigui County, Three Gorges area of Yangtze River, is sweet and crisp, which is well liked by consumers. Therefore, it was selected to investigate the effect of different harvesting time on the quality of orange dark tea in this study.

### 3.3. Effects of Plucking Time on Quality of Peach Leaf Orange Dark Tea

#### 3.3.1. Effects of Harvesting Time on Sensory Quality

Table 2 and Table S3 display the organoleptic analysis results of orange dark teas processed with Peach leaf oranges harvested at various time periods. The fruit ripeness increases with the delay of plucking time. Despite no discernible impact on infusion color or aroma, picking time does have a substantial impact on the flavor quality of the Peach leaf orange dark tea. This dark tea has a brilliant reddish infusion color, and a sweet pleasant scent to well coordinate the fruit and tea aroma. In terms of acidity, Peach leaf orange dark teas are slightly sour when processed with August-collected Oranges (*Citrus × sinensis*). With the delay of harvesting time, the scores of sweetness, bitterness, astringency and coordination showed an uptrend first and then a downtrend, with peak value appearing in September, indicating that the orange tea processed with September-harvested Peach leaf oranges has a sweeter flavor, lighter bitterness and astringency, and a coordinated flavor. The lowest score was observed in August, followed by December, indicating that the orange dark teas processed with Oranges (*Citrus × sinensis*) harvested in these two months do not have a sufficiently sweet and mellow flavor, but a heavy bitter and astringent flavor, leading to poor flavor coordination. In the total score, it was also shown that with the delay of harvesting time, the sensory score followed a trend of increasing first and then decreasing, with the maximum value emerging in September. This further indicates that September could be the most suitable time for harvesting Peach leaf oranges for processing dark tea, endowing the orange dark tea with optimal overall quality, such as fruit and tea aroma, sweet and mellow taste, and a harmonious balance between tea flavor and fruit aroma. It can also be seen that neither premature (August) nor belated (December) harvesting periods are suitable for processing Peach leaf oranges into citrus dark tea.

#### 3.3.2. Effects of Harvesting Time on Quality Composition of Flavor

The main quality components in the peel and tea of Peach leaf orange dark teas at different harvesting time periods were shown in Table 3 and Table 4. As shown in Table 3, the main active constituents in the peel were hesperidin (38.05–43.65 mg/g), polyphenols (24.9–27.3 mg/g), and flavonoids (4.53–10.2 mg/g). With the delay of harvesting time, the peel showed a downtrend in soluble protein, flavonoids, polyphenols, hesperidin, synephrine, and limonin, with the greatest decrease in synephrine and flavonoids, reaching 55.8% and 55.6%, respectively. Comparatively, the peel showed insignificant changes in polyphenols, hesperidin, synephrine, and limonin between August and September, and soluble proteins, polyphenols, flavonoids, polyphenols, hesperidin, and limonin between November and December. In Table 3, it was also shown that unlike the other substances, polysaccharides had a content increase of 27.7% with the delay of harvesting time. The above peel analysis results showed that with the delay of harvesting time, the content of polysaccharides increased significantly (*p* < 0.05), while the other substances decreased significantly (*p* < 0.05).

Plucking time had some impact on the quality components of orange dark tea, but the effect degree was not consistent. With the delay of harvesting time, the tea showed slightly irregular changes in polyphenols, amino acids and theaflavins, while a significant increase in soluble sugar content (Table 4).

The content of substances in the Peach leaf orange peel showed a certain regularity with the delay of harvesting time or with increasing maturity. As previously reported, the increase in polysaccharide content with fruit maturity facilitated hydrolysis during processing to produce sweet substances such as monosaccharides and disaccharides, which in turn can enhance the sweet and mellow flavor of orange dark tea [24,25]. Flavonoids, hesperidin, synephrine, and limonin in citrus fruits are important contributors to bitterness and astringency, especially limonin, which is the most important bitterness-causing substance [26]. The decrease in the content of these substances with increasing harvesting time in this experiment contributed to the reduction in bitter and astringent flavor of orange dark tea [27], which was well supported by the sensory evaluation results.

#### 3.3.3. Effects of Harvesting Time on Aroma Compounds

The HPME-GC-MS technique was used to analyze the aroma of orange dark teas processed with Oranges (*Citrus × sinensis*) of different maturity and the results were shown in Table S4. A total of 111 compounds were identified, including 35 aldehydes and ketones, 27 alcohols, 25 olefins, 9 acids and esters, and 14 other compounds, with their content and proportion shown in Table 5. The content of the major substances followed the order of alcohols, aldehydes and ketones, and olefins. With the delay of harvesting time, the Peach leaf oranges became more mature, and a downtrend was observed in total volatile components as well as the content of each listed category, but the decrease in content was not obvious after September. In terms of substance proportion (Table 5), it was shown that with the delay of harvesting time, the proportion of alcohols gradually decreased from August to October, but increased from November to December. Meanwhile, aldehydes and ketones, and acid esters showed a trend of increasing first and then decreasing, reaching their maximum values in November.

**Figure 3:**
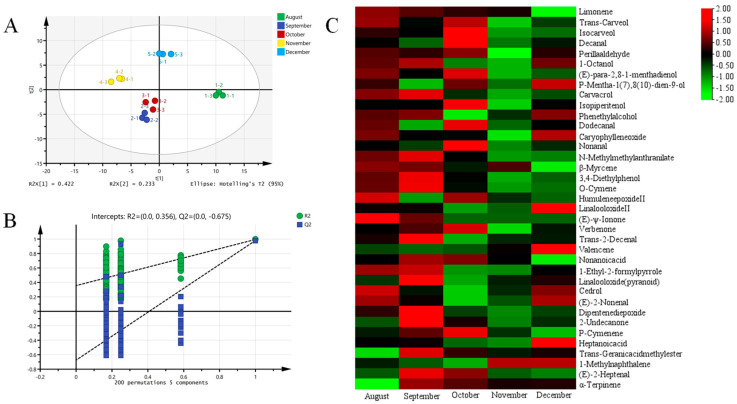
Effect of harvesting time on the content of volatile compounds in orange dark teas processed with Oranges (*Citrus × sinensis*) harvested from August to December. (**A**) PLS-DA score chart. (**B**) Permutation inspection. (**C**) Thermal map for the content of differential volatile compounds.

PLS-DA analysis was conducted to investigate variations in volatile components in orange dark teas processed with Oranges (*Citrus × sinensis*) of varying maturity (Figure 3A,B). The five groups of orange dark teas could be well distinguished by the PLS-DA model (R2x = 0.916, R2y = 0.989, Q2 = 0.962). The September and October samples showed similar volatile compounds, in contrast to notable differences in volatile compounds from August, November, and December samples. A total of 37 distinct aroma components were identified in the five groups of orange dark teas based on variable importance in projection (VIP) > 1 and *p* < 0.05, including 8 alcohols, 7 aldehydes, 6 olefins, 3 ketones, 2 phenols, 2 esters, 2 acids, and 7, and variations in their contents are displayed in Figure 3C and Table S5. With the delay of harvesting time, a downtrend was observed in the content of limonene, trans-carveol, β-myrcene, humulene epoxide II, trans-2-decenal and 1-ethyl-2-formylpyrrole, especially after October. Meanwhile, December samples showed a significant increase in the content of p-mentha-1 (7), 8 (10)-dien-9-ol, linalool oxide II and heptanoic acid. Additionally, caryophyllene oxide and valencene remained relatively stable from August to October, but increased significantly thereafter, with a slight increase in 1-methylnaphthalene in the later harvest period. From August to December, despite a slight fluctuation, 1-octanol and phenethyl alcohol were relatively stable; the levels of (E)-ψ-ionone decreased steadily to undetectable levels after October; and α-terpinene appeared from September onward and decreased slightly later in the harvest season. Other substances showed an uptrend first and then a downtrend with the delay of harvesting time, with most substances reaching their maximum levels in September or October.

Based on the volatile composition analysis results, the total amount of each aroma substance decreased with increasing harvesting time. Among them, limonene is a representative aroma substance in citrus fruits, which can account for the strong floral and fruity aroma of citrus fruits, and its chemical nature is more active, allowing it to be easily converted to limonene oxides, carvacrol, and other substances through degradation and oxidation [28]. Meanwhile, limonene has a distinct lemon smell and its content decrease may be related to the decrease in freshness in orange dark tea. Furthermore, specific aroma compounds detected in tea samples produced from August and September harvested Oranges (*Citrus × sinensis*) showed progressive reduction to undetectable levels with advancing maturity. These included fresh herbal aldehydes such as heptanal and sabinene, along with thymol, decanoic acid, 1,6-dihydrocarveol, and phenanthrene. This compositional change was strongly associated with diminished freshness attributes. Moreover, there are some substances only detectable in the December orange dark tea, such as farnesol, α-campholenal, sabina ketone, nootkatone, geranyl acetate, and eugenol. Among them, farnesol and geranyl acetate are substances with floral and fruity characteristics, and they may be related to the sweet and fruity aroma of ripe orange tea [29].

#### 3.3.4. Effects of Harvesting Time on Biological Activities

*(i)*  *Antioxidant activity*

Orange dark tea is composed of Peach leaf orange peel and tea, and antioxidant activity is its important bioactivity. Therefore, this paper also analyzed the antioxidant activity of peel at different harvesting periods, and the results were shown in Table 6. With the delay of harvesting time, a downtrend was observed in the FRAP ferric ion reducing capacity, as well as the scavenging capacity of DPPH and ABTS free radicals. The FRAP ferric ion reducing capacity showed a slight but not significant difference between August and September and remained stable between October and December. The DPPH radical scavenging capacity was the highest in August orange peel (29.7 μmol TE/g) and showed no significant difference in the orange peels collected from September to December. The scavenging capacity of ABTS radicals was significantly higher in peels collected in August and September than in those collected in other months, but with no significant difference between August and September or between October and December peels. This suggests that only peels collected in August and September have the strongest antioxidant activity.

The impact of plucking time on the antioxidant content of Peach leaf orange tea was also investigated (Table 6). Plucking time was found to have no significant effect on either the FRAP iron ion reduction capacity or the ABTS radical scavenging capacity of the orange tea. Nonetheless, it had a major impact on the DPPH free radical scavenging ability, with the highest antioxidant capacity (380.41 μmol TE/g) for October-orange tea, and no significant difference among the other four orange teas in antioxidant capacity. In vitro antioxidant assays cannot be directly compared due to their different methods and principles, so we introduced the antioxidant potency composite (APC) index for a comprehensive antioxidant evaluation [30]. By calculating the APC values of orange teas processed with Oranges (*Citrus × sinensis*) harvested at different time periods (Table 7), the three-indicator APC indexes were found to be the highest for APC-FRAP in August, APC-ABTS in September and APC-DPPH in October. The APC value of the combined antioxidant capacity index was the highest in October (99.58), followed by December (99.11) and August (99.10), which were closely similar to each other, and then by September (98.31) and November (98.40). This suggests that the highest integrated antioxidant capacity could be in the October-orange dark tea.

*(ii)*  *Ability to inhibit activity of key enzymes in glycolipid metabolism*

The primary enzymes involved in metabolism of glycolipids in the human body are α-amylase and α-glucosidase [31]. Among them, α-amylase, a digestive enzyme released by the pancreas and salivary glands, performs the primary function of assisting the body in breaking down sugars and preserving stable blood glucose levels, which in turn helps intestinal digestion and absorption of food [32]. The primary function of α-glucosidase, a crucial hydrolytic enzyme in the human body, is to hydrolyze monosaccharides and facilitate the body to absorb glucose from food [33]. The results of the inhibitory effects of orange tea processed at different harvesting times on α-glucosidase and α-amylase activities were shown in Table 8. When the enzyme activity is 50% inhibited, the IC_50_ value shows the sample concentration needed; the lower the IC_50_ value, the stronger the inhibitory impact [22]. The IC_50_ values for the inhibitory capacity of Peach leaf orange teas on α-glucosidase and α-amylase demonstrated a noteworthy upward trend with the delay of harvesting time, suggesting that the inhibitory effect on both enzymes diminished with the delay of harvesting time.

Analysis of the IC_50_ values revealed that the IC_50_ of Peach leaf orange dark tea extract for inhibiting α-glucosidase and α-amylase activities was above 300 μg/mL and 400 μg/mL, respectively. Generally, acarbose is commonly used as a positive control in anti-diabetic drug evaluations [34]. According to literature reports, the IC50 values of acarbose for inhibiting α-glucosidase and α-amylase activities were 35.52 ± 1.23 μg/mL and 50.01 ± 0.92 μg/mL, respectively [35]. This indicated that the inhibitory effect of Peach leaf orange dark tea extract on both α-glucosidase and α-amylase activities was significantly weaker than that of acarbose. However, since the extract is rich in natural bioactive compounds such as tea polyphenols, flavonoids, and synephrine, long-term consumption is free from side effects [36,37]. Therefore, it can be safely utilized as a functional food or dietary supplement ingredient, contributing to the auxiliary regulation of glucose and lipid metabolism in humans [38].

*(iii)*  *Correlation analysis between bioactivity and functional components of Peach leaf orange dark teas under different orange plucking time conditions*

**Figure 4:**
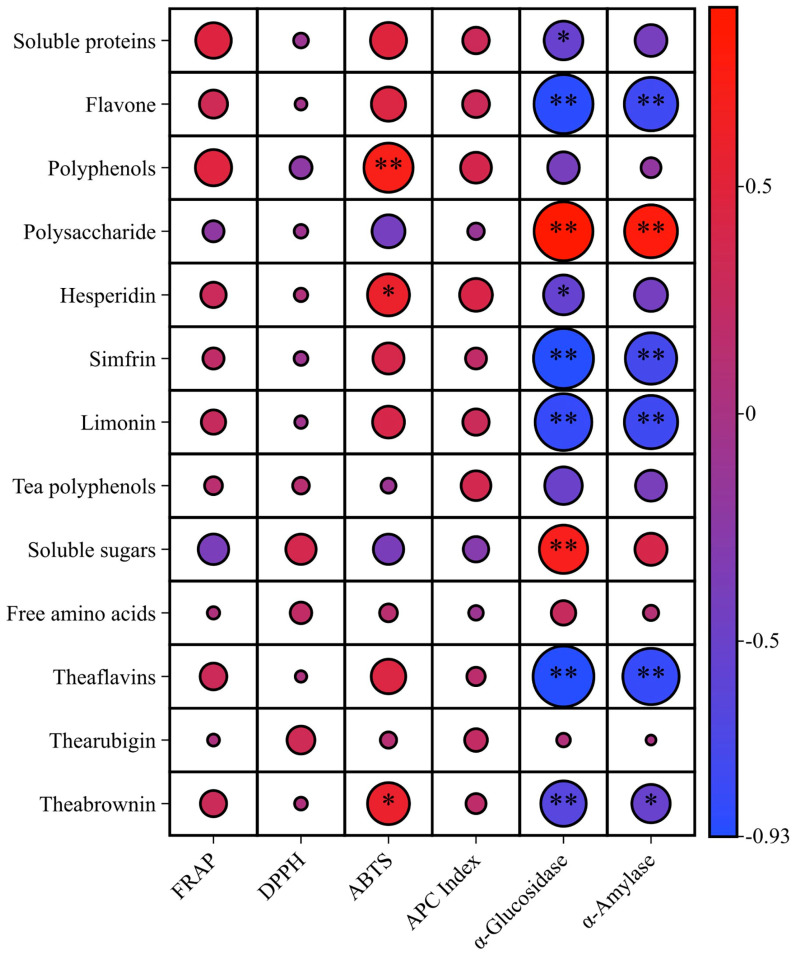
Correlation analysis between main functional components and biological activities of orange dark teas processed with Peach leaf oranges of different maturity. Note: The Pearson correlation coefficients are shown in each cell. Asterisks indicate statistical significance (*, *p* < 0.05, **, *p* < 0.01).

To further analyze the impact of bioactive components in Peach Leaf Orange Tea on its functionality, this study also examined the correlation between the non-volatile components and bioactivities of Peach Leaf Orange Tea harvested at different times. The results were presented in Figure 4. With the exception of polysaccharides, soluble sugars, and free amino acids, all other components showed a positive correlation with FRAP (Ferric Reducing Antioxidant Power) inhibition capacity. Substances with a correlation coefficient greater than 0.4 included tea polyphenols, soluble proteins, thearubigins, and limonin. Regarding ABTS radical scavenging activity, with the exception of polysaccharides, soluble sugars, tea polyphenols, free amino acids, and thearubigins, all other components exhibited a positive correlation with ABTS inhibition capacity. Notably, the contents of polyphenols, hesperidin, and theabrownins also showed a significantly positive correlation with ABTS radical scavenging capacity (*p* < 0.05).

With the exception of polysaccharides, soluble sugars, free amino acids, and thearubigins, the content of all other substances showed a negative correlation with the IC_50_ value of α-glucosidase inhibition. Among them, the effects of flavonoids, polysaccharides, synephrine, limonin, theaflavins, and theabrownins reached a highly significant level (*p* < 0.01), indicating that these substances contribute substantially to the inhibition of α-glucosidase. Similarly, the correlation analysis with the IC_50_ value of α-amylase yielded comparable results. Again, excluding polysaccharides, soluble sugars, free amino acids, and thearubigins, the α-amylase IC_50_ value also exhibited a negative correlation with the content of the other substances. The correlations with the content of flavonoids, polysaccharides, synephrine, limonin, and theaflavins reached a significant level (*p* < 0.01), likewise demonstrating that these compounds contribute significantly to the inhibition of α-amylase.

This experiment demonstrated that the contents of theabrownins, polyphenols, hesperidin, and other substances in Peach Leaf Orange black tea were significantly correlated with ABTS radical scavenging capacity. Theabrownins exhibit strong ABTS radical scavenging activity, and their mechanism of action may involve inhibiting reactive oxygen species formation by suppressing xanthine oxidase [39,40,41]. Polyphenols and hesperidin in citrus peel have also been confirmed to possess strong antioxidant activity. Phenolic compounds likely exert their antioxidant effects by donating protons or electrons, thereby protecting cells from free radical damage [42]. Meanwhile, hesperidin enhances cellular antioxidant defense by eliminating free radicals and reactive oxygen species [43]. Since fruit maturity affects the content of active substances, it leads to variations in antioxidant activity. For example, a study by Singh B. found that as Jambolan (*Syzygium cumini*) maturity increases, the content of bioactive compounds such as polyphenols, flavonoids, and hesperidin in the peel decreased, resulting in a corresponding reduction in ABTS and DPPH radical scavenging capacities [44].

Correlation analysis also showed that the inhibitory effects of orange dark tea on α-glucosidase and α-amylase were significantly influenced by flavonoids, synephrine, limonin, theaflavins, etc. Synephrine may have an inhibitory effect by binding to the enzyme active site or by altering the conformation of the enzyme molecule [45]. Several studies have demonstrated that flavonoids can inhibit enzyme activity by forming complexes and inducing conformational changes [46]. Although the precise mechanism remains unclear, limonin, a significant citrus acyl compounds with a furan ring structure, may be crucial to the inhibitory activity of α-glucosidase [47]. As phenolic compounds that attach to proteins via covalent bonds, hydrogen bonds, and hydrophobic interactions, the flavones can influence the structure and characteristics of proteins [48]. Research by Li et al. revealed that theaflavins exhibited potent α-amylase inhibitory activity, with efficacy following a distinct structure-dependent hierarchy: TF3 (heaflavin-3, 3′-digallate) > TF2B (theaflavin-3′-gallate) > TF2A (theaflavin-3-gallate) > TF1 (theaflavin). The inhibition mechanism involves specific hydrophobic interactions between theaflavins and hydrophobic pockets adjacent to the enzyme’s active site. This binding induces conformational alterations in α-amylase’s secondary structure, resulting in complete loss of enzymatic activity [49]. Furthermore, theaflavins form stable monomeric complexes with α-glucosidase through hydrogen bonding and van der Waals interactions. This binding induces conformational restructuring of the enzyme, thereby mediating potent inhibitory effects on α-glucosidase activity [50]. This study further revealed that the inhibitory efficacy of orange tea against both α-glucosidase and α-amylase progressively decreased with advancing fruit maturity. This trend paralleled the reduction in flavonoid content, aligning with established literature. For instance, Li et al. demonstrated that fluctuations in citrus flavonoids exhibited a positive correlation with their inhibitory potency toward α-glucosidase and α-amylase activities [37].

## 4. Conclusions

The citrus variety significantly influences the quality of citrus dark tea. Among them, Oranges (*Citrus × sinensis*) are the most suitable for processing into dark tea, followed by Mandarins (*Citrus × reticulata*). Grapefruits (*Citrus maxima*) are the least suitable, while most Lemons (*Citrus × limon*) and Tangerines (*Citrus × reticulata*) are also not well-suited for this purpose. Additionally, harvesting time has a significant effect on the flavor quality of the Peach leaf orange dark tea, but no significant impact on its infusion color and aroma. September has been shown to be the best harvesting period for Peach leaf orange, and the processed Peach leaf orange dark tea not only had a strong fruity aroma, sweet and mellow taste, and well-balanced tea and fruit aroma, but also exhibited better antioxidant capacity and inhibitory effect on key enzymes of glycolipid metabolism. This study has laid a scientific basis for selection of citrus varieties and harvesting period of citrus fruits for processing citrus dark tea, which may contribute to the development of related industries.

## Figures and Tables

**Table 1:** Average scores and correlation analysis of sensory indicators for citrus dark teas made from different types of citrus fruits.

		M	T	O	G	L	P M-T	P M-O	P M-G	P M-L	P T-O	P T-G	P T-L	PO-G	P O-L	P G-L
Soup color	Score	8.93	8.90	9.09	8.91	8.97	0.21	<0.01	0.62	0.08	<0.01	0.43	0.08	<0.01	<0.01	0.03
Aroma	Score	22.47	20.78	22.77	22.22	23.87	<0.01	0.26	0.36	<0.01	<0.01	<0.01	<0.01	0.06	<0.01	<0.01
	Subdivide	14.44	13.83	14.44	14.25	14.93	<0.01	0.94	0.00	<0.01	<0.01	<0.01	0.01	<0.01	<0.01	<0.01
	Coordination	8.03	7.80	8.33	7.98	8.93	<0.01	<0.01	0.15	<0.01	<0.01	<0.01	<0.01	<0.01	<0.01	<0.01
Taste	Score	28.98	27.41	30.19	22.56	26.07	<0.01	<0.01	<0.01	<0.01	<0.01	<0.01	<0.01	<0.01	<0.01	<0.01
	Sour	5.51	5.46	5.45	5.26	4.67	0.26	0.16	<0.01	<0.01	0.74	<0.01	<0.01	<0.01	<0.01	<0.01
	Sweet	6.08	6.19	6.06	4.86	5.67	-	-	-	-	-	-	-	-	-	-
	Bitter	5.76	6.16	6.44	4.29	5.40	<0.01	<0.01	<0.01	<0.01	<0.01	<0.01	<0.01	<0.01	<0.01	<0.01
	Astringent	5.96	5.78	6.24	4.25	5.67	0.03	<0.01	<0.01	<0.01	<0.01	<0.01	0.04	<0.01	<0.01	<0.01
	Taste harmony	5.67	5.54	6.00	3.90	4.67	<0.01	<0.01	<0.01	<0.01	<0.01	<0.01	<0.01	<0.01	<0.01	<0.01
	Score	60.38	57.09	62.05	53.70	58.90	<0.01	<0.01	<0.01	<0.01	<0.01	<0.01	0.01	<0.01	<0.01	<0.01

Note: M, Mandarin; T, Tangerine; O, Orange; G, Grapefruit; L, Lemon; P, *p* values; M-T, mandarin versus tangerine; M-O, mandarin versus orange; M-G, mandarin versus grapefruit; M-L, mandarin versus lemon; T-O, tangerine versus orange; T-G, tangerine versus grapefruit; T-L, tangerine versus lemon; O-G, orange versus grapefruit; O-L, orange versus lemon; G-L, grapefruit versus lemon.

**Table 2:** Sensory scores of orange dark teas processed with Peach leaf oranges harvested at different time periods.

Harvest Time	Soup Color	Aroma		Taste					Score
		Aroma	Coordination	Sour	Sweet	Bitter	Astringent	Coordination	
August	9 ± 0.1 ^a^	15 ± 0.1 ^a^	9 ± 0 ^a^	5.7 ± 0.6 ^a^	6 ± 0 ^b^	6 ± 0 ^b^	5.7 ± 0.2 ^b^	5.8 ± 0.4 ^b^	62.1 ± 0.8 ^c^
September	9 ± 0.1 ^a^	14.9 ± 0.1 ^a^	8.9 ± 0.1 ^a^	6 ± 0.3 ^a^	7 ± 0 ^a^	7.2 ± 0.4 ^a^	7.2 ± 0.4 ^a^	7 ± 0 ^a^	67.2 ± 0.6 ^a^
October	8.9 ± 0.1 ^a^	14.9 ± 0.1 ^a^	8.9 ± 0.1 ^a^	5.9 ± 0.2 ^a^	6.1 ± 0.2 ^b^	6.1 ± 0.9 ^b^	6.2 ± 0.8 ^a^	6.1 ± 0.7 ^b^	63.2 ± 1.8 ^bc^
November	9 ± 0.1 ^a^	15 ± 0.1 ^a^	9 ± 0 ^a^	6.3 ± 0.6 ^a^	6.1 ± 0.17 ^b^	6.2 ± 0.7 ^b^	6.3 ± 0.6 ^a^	6.3 ± 0.4 ^b^	64.2 ± 1.2 ^b^
December	9 ± 0.1 ^a^	14.9 ± 0.2 ^a^	8.9 ± 0.1 ^a^	6.1 ± 0.6 ^a^	6 ± 0 ^b^	5.8 ± 0.3 ^b^	6 ± 0 ^a^	6.0 ± 0.6 ^b^	62.8 ± 1.2 ^c^

Note: Multiple comparisons were performed using the LSD method, and different lowercase letters in the same column indicate significant differences at *p* < 0.05.

**Table 3:** Physicochemical analysis of peels at different maturity levels (mg/g).

Harvest Time	August	September	October	November	December
Soluble proteins	2.96 ± 0.54 ^a^	2.66 ± 0.21 ^b^	2.4 ± 0.19 ^bc^	2.35 ± 0.08 ^c^	2.5 ± 0.12 ^bc^
Flavone	10.2 ± 1 ^a^	8.79 ± 0.88 ^b^	8.28 ± 0.78 ^b^	5.47 ± 0.89 ^c^	4.53 ± 1.53 ^c^
Polyphenols	26.5 ± 1.1 ^ab^	27.3 ± 0.9 ^a^	25.7 ± 1.9 ^bc^	24.9 ± 0.4 ^c^	25.6 ± 1.0 ^bc^
Polysaccharide	30 ± 1.9 ^b^	31.2 ± 2.8 ^b^	31.2 ± 2.9 ^b^	36.9 ± 4.2 ^a^	38.3 ± 2.1 ^a^
Hesperidin	43.65 ± 3.62 ^a^	45.83 ± 3.18 ^a^	44.81 ± 4.35 ^a^	38.82 ± 4.78 ^b^	38.05 ± 4.07 ^b^
Simfrin	0.86 ± 0.08 ^a^	0.83 ± 0.05 ^a^	0.71 ± 0.04 ^b^	0.45 ± 0.05 ^c^	0.38 ± 0.04 ^d^
Limonin	2.02 ± 0.3 ^a^	1.33 ± 0.26 ^b^	1.27 ± 0.29 ^b^	0.97 ± 0.08 ^c^	0.9 ± 0.04 ^c^

Note: Multiple comparisons were performed using the LSD method, and different lowercase letters in the same row indicate significant differences at *p* < 0.05.

**Table 4:** The content of tea quality components (%) and water-soluble pigments (mg/g) in tea at different harvesting time intervals.

Harvest Time	Tea Polyphenols (%)	Free Amino Acids (%)	Soluble Sugars (%)	Theaflavins (mg/g)	Thearubigins (mg/g)	Theabrownins (mg/g)
August	9.00 ± 0.15 ^a^	1.71 ± 0.03 ^ab^	3.45 ± 0.11 ^c^	0.44 ± 0.01 ^a^	8.37 ± 0.63 ^a^	21.49 ± 0.40 ^ab^
September	8.75 ± 0.19 ^bc^	1.68 ± 0.04 ^b^	3.38 ± 0.09 ^c^	0.43 ± 0.02 ^a^	7.74 ± 0.21 ^a^	22.75 ± 0.11 ^a^
October	8.76 ± 0.17 ^bc^	1.74 ± 0.06 ^a^	3.62 ± 0.08 ^b^	0.42 ± 0.04 ^a^	8.39 ± 0.99 ^a^	22.22 ± 1.07 ^ab^
November	8.61 ± 0.11 ^c^	1.72 ± 0.01 ^a^	3.75 ± 0.09 ^a^	0.38 ± 0.01 ^b^	8.15 ± 0.84 ^a^	21.62 ± 1.01 ^ab^
December	8.81 ± 0.25 ^b^	1.73 ± 0.02 ^a^	3.66 ± 0.04 ^b^	0.47 ± 0.04 ^a^	8.50 ± 1.10 ^a^	21.26 ± 0.98 ^b^

Note: Multiple comparisons were performed using the LSD method, and different lowercase letters in the same column indicate significant differences at *p* < 0.05.

**Table 5:** Content and proportion of aroma types in orange dark teas processed with Oranges (*Citrus × sinensis*) harvested at different time periods (μg/g).

Class	Assay (μg/g)					Proportion (%)				
	August	September	October	November	December	August	September	October	November	December
Alcohols	584.48 ± 47.69 ^a^	484.54 ± 19.47 ^bc^	488.73 ± 29.58 ^b^	356.02 ± 20.52 ^d^	401.72 ± 30.53 ^cd^	39.32	43.47	38.12	24.87	38.07
Aldehydes and ketones	352.28 ± 31.8 ^a^	333.97 ± 13.98 ^ab^	355.5 ± 22.46 ^a^	289.84 ± 16.48 ^bc^	268.2 ± 20.07 ^c^	23.70	28.98	26.28	17.85	27.69
Olefins	364.97 ± 18.89 ^a^	298.16 ± 37.98 ^b^	309.02 ± 17.84 ^ab^	287.34 ± 20.17 ^bc^	228.74 ± 19.81 ^c^	24.55	17.22	23.46	48.51	24.07
Esters	85.89 ± 4.24 ^a^	61.04 ± 1.96 ^b^	48.27 ± 4.14 ^c^	37.71 ± 3.54 ^d^	42.07 ± 3.56 ^cd^	5.78	3.87	4.80	2.51	3.76
Others	99 ± 7.1 ^a^	93.29 ± 4.89 ^ab^	82.18 ± 5.38 ^b^	62.69 ± 5.21 ^c^	67.4 ± 7.8 ^c^	6.66	6.47	7.34	6.25	6.40
Total	1486.62 ± 109.71 ^a^	1271 ± 78.29 ^b^	1283.69 ± 79.39 ^b^	1033.6 ± 65.92 ^c^	1008.13 ± 81.76 ^c^	100.00	100.00	100.00	100.00	100.00

Note: Multiple comparisons were performed using the LSD method, and different lowercase letters in the same row indicate significant differences at *p* < 0.05.

**Table 6:** Antioxidant activity of orange peels and orange dark teas at different harvesting time periods (μmol TE/g).

Harvest Time	Orange Peel			Orange Tea		
	FRAP	DPPH	ABTS	FRAP	DPPH	ABTS
August	34.93 ± 0.61 ^a^	29.7 ± 1.3 ^a^	118.91 ± 1.57 ^a^	267.99 ± 3.82 ^a^	370.31 ± 4.15 ^b^	325.15 ± 0.68 ^a^
September	33.26 ± 1.71 ^ab^	23.91 ± 3.14 ^b^	118.16 ± 2.04 ^ab^	264.63 ± 3.85 ^a^	365.86 ± 4.86 ^b^	325.29 ± 0.7 ^a^
October	32.08 ± 0.05 ^b^	23.59 ± 1.99 ^b^	116.2 ± 0.52 ^bc^	264.83 ± 2.67 ^a^	380.41 ± 1.6 ^a^	325.06 ± 1.95 ^a^
November	31.66 ± 0.44 ^b^	22.67 ± 0.49 ^b^	115.52 ± 1.42 ^c^	261.46 ± 3.74 ^a^	372.57 ± 4.97 ^b^	324.28 ± 1.45 ^a^
December	32.02 ± 1 ^b^	22.55 ± 0.74 ^b^	115.42 ± 0.96 ^c^	267.86 ± 5.61 ^a^	371.15 ± 4.38 ^b^	324.66 ± 1.12 ^a^

Note: Multiple comparisons were performed using the LSD method, and different lowercase letters in the same column indicate significant differences at *p* < 0.05. The final results are expressed as Trolox equivalents (TE) in terms of μmol TE per gram of each sample (μmol TE/g).

**Table 7:** Antioxidant potency composite (APC) index for comprehensive antioxidant activity of orange dark teas processed with Peach leaf oranges of different maturity.

Harvest Time	FRAP APC	DPPH APC	ABTS APC	Synthesis APC
August	100.00	97.35	99.96	99.10
September	98.75	96.18	100.00	98.31
October	98.82	100.00	99.93	99.58
November	97.56	97.94	99.69	98.40
December	99.95	97.57	99.81	99.11

Note: FRAP, Ferric Reducing Antioxidant Power; DPPH, 2, 2-Diphenyl-1-picrylhydrazyl; ABTS, 2, 2′-Azino-bis (3-ethylbenzothiazoline-6-sulfonic acid).

**Table 8:** Effect of harvesting time on inhibition of α-glucosidase and α-amylase activity in orange dark tea (IC_50_ ug/mL).

Harvest Time	α-Glucosidase	α-Amylase
August	304.7 ± 5.24 ^d^	471.95 ± 9.09 ^e^
September	339.4 ± 9.49 ^c^	496.23 ± 9.99 ^d^
October	346.36 ± 10.19 ^c^	692.13 ± 7.64 ^c^
November	374.79 ± 1.76 ^b^	764.4 ± 4.88 ^b^
December	388.35 ± 5.51 ^a^	790.66 ± 2.07 ^a^

Note: Multiple comparisons were performed using the LSD method, and different lowercase letters in the same column indicate significant differences at *p* < 0.05.

## Data Availability

The original contributions presented in this study are included in the article/supplementary material. Further inquiries can be directed to the corresponding authors.

## Supplementary Materials

The following supporting information can be downloaded at: https://www.mdpi.com/article/10.3390/foods14183181/s1, Figure S1. Scores for infusion color, aroma, taste, and total sensory score. (A) Infusion color score. (B) Aroma score. (C) Taste score. (D) Total sensory score; Table S1: Botanical nomenclature and common names of 74 citrus varieties; Table S2: Sensory evaluation results of different varieties of citrus dark tea; Table S3: Sensory quality of Peach leaf orange dark tea at different harvest times; Table S4: Analysis of volatile components in orange tea with different maturity levels (μg/g); Table S5: Differential aroma component content of orange dark tea at different harvest times (μg/g).

## Abbreviations

The following abbreviations are used in this manuscript:

BSA	bovine serum albumin
ABTS	2, 2′-azinobis (3-ethylbenzothiazoline-6-sulfonic acid)
FRAP	Ferric Reducing Antioxidant Power
DPPH	2, 2-Diphenyl-1-picrylhydrazyl
HPLC	High Performance Liquid Chromatography
HS-SPME	Headspace Solid-Phase Microextraction
GC-MS	Gas Chromatography-Mass Spectrometry
DVB	Divinylbenzene
CAR	Carboxen
PDMS	Polydimethylsiloxane
EI	Electron Impact Ionization
TE	Trolox equivalents
LSD	Least Significant Difference
IC_50_	Half Maximal Inhibitory Concentration
VIP	Variable Importance in Projection
APC	Antioxidant Potency Composite
ROS	Reactive Oxygen Species
TF1	Theaflavin
TF2A	theaflavin-3-gallate
TF2B	theaflavin-3′-gallate
TF3	theaflavin-3, 3′-digallate
