# Supplementary material for: Effects of Different Citrus Varieties and Harvesting Time on the Quality of Citrus Dark Tea
Source: Foods. 2025 Sep 12;14(18):3181. doi: 10.3390/foods14183181 (PMC12469959; doi:10.3390/foods14183181)
Supplement: Supplementary file 1 [file foods-14-03181-s001.zip › foods-3847047-supplementary.pdf]

Supplementary Materials:

Figure_S1. Scores for infusion color, aroma, taste, and total sensory score. (A) Infusion color score. (B) Aroma score. (C) Taste score. (D) Total sensory score.

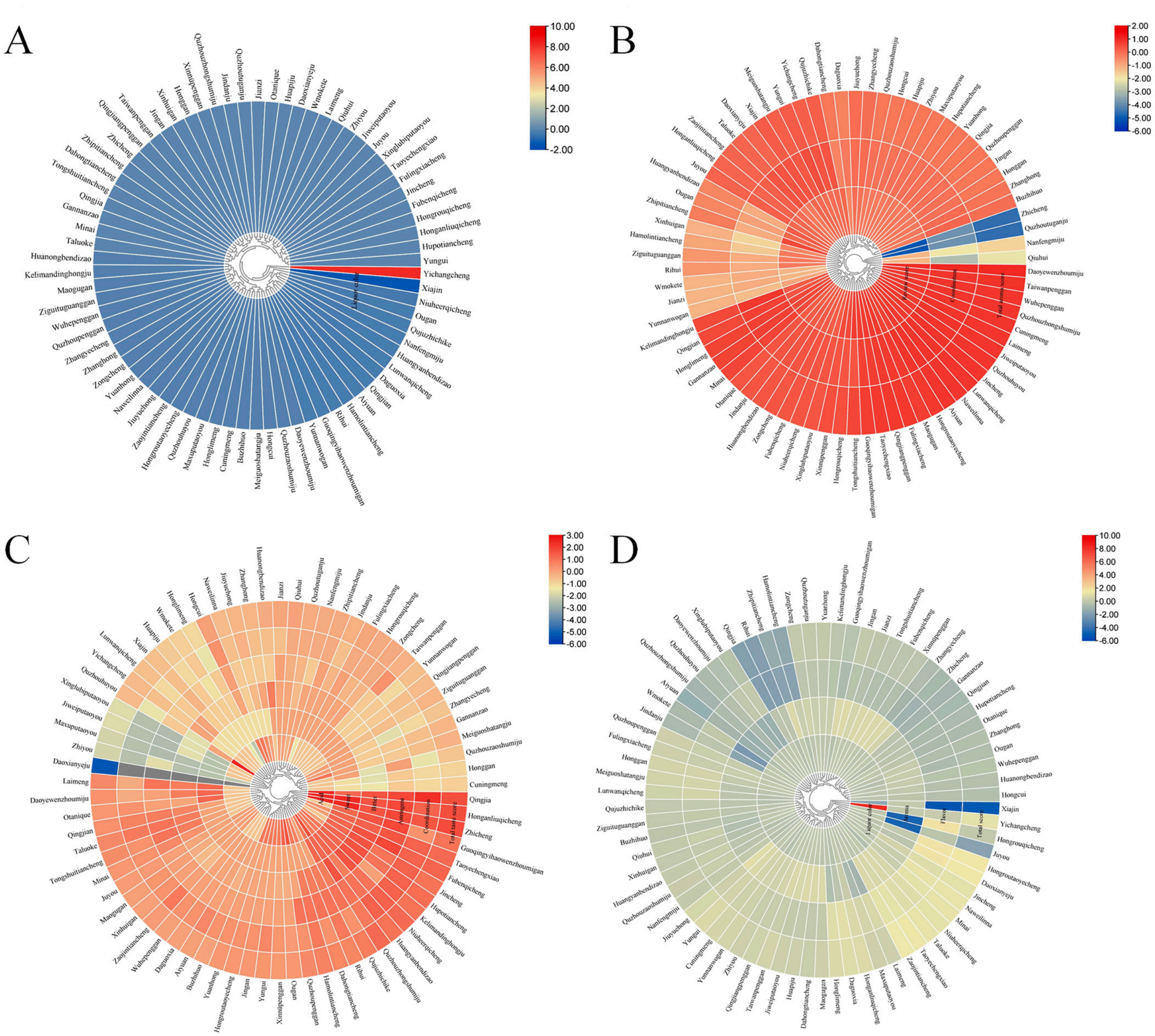

Table_S1 Botanical nomenclature and common names of 74 citrus varieties

Common Name	Botanical Name
Red dark willow navel orange	*Citrus sinensis*
Qingjia navel orange	*Citrus sinensis*
Quzhou medium-ripe oranges	*Citrus unshiu*
Peach leaf orange	*Citrus sinensis*
Fukumoto navel orange	*Citrus sinensis*
Golden orange	*Citrus sinensis*
Newhall	*Citrus sinensis*
National Day No. 1 Wenzhou mandarin	*Citrus unshiu*
Klimantin red oranges	*Citrus clementina*
Early Golden Sweet Orange	*Citrus sinensis*
Amber sweet orange	*Citrus sinensis*
Trifoliate orange	*Poncirus trifoliata*
Qu tangerine	*Citrus reticulata*
Lime	*Citrus aurantiifolia*
Huangyan is local early	*Citrus reticulata*
Inaba Wenzhou mandarin	*Citrus unshiu*
72-1 copper water sweet orange	*Citrus sinensis*
Kiyomi	*Citrus unshiu* × *sinensis*
Taroko	*Citrus sinensis*
Red-fleshed Peach leaf orange	*Citrus sinensis*
Big red sweet orange	*Citrus sinensis*

Minaxia orange	*Citrus sinensis*
Daoxian Wild Mandarin	*Citrus reticulata Blanco var. daoxianensis*
Quzhou mandarin orange	*Citrus reticulata*
Ehime	*Citrus reticulata*
Ortanic	*Citrus reticulata*
Seedless ponkan	*Citrus reticulata*
Hamorim sweet orange	*Citrus sinensis*
Xinnv ponkan	*Citrus reticulata*
Maogu mandarin	*Citrus reticulata*
Yungui orange	*Citrus sinensis*
Sunshine orange	*Citrus reticulata*
Voling summer orange	*Citrus sinensis*
Yuanhong	*Citrus sinensis*
Orange grapefruit	*Citrus reticulata* × *paradisi*
Mandarin oranges	*Citrus reticulata*
Citrus aurantium	*Poncirus trifoliata* × *Citrus sinensis*
Red-fleshed navel orange	*Citrus sinensis*
Taiwan ponkan	*Citrus reticulata*
Brown orange	*Citrus sinensis*
Huanong local early	*Citrus reticulata*
Ignorant fire	*Citrus reticulata* × *sinensis*
Red crisp navel orange	*Citrus sinensis*

Large-fruited summer orange	*Citrus sinensis*
Gannan early	*Citrus sinensis*
Grapefruit	*Citrus paradisi* × *reticulata*
Navelina navel orange	*Citrus sinensis*
Qingjiang mandarin	*Citrus reticulata*
Thin-peel sweet orange	*Citrus sinensis*
American granulated sugar oranges	*Citrus reticulata*
Lun night	*Citrus sinensis*
Xinhui mandarin	*Citrus reticulata*
September red navel orange	*Citrus sinensis*
Yichang orange	*Citrus ichangensis*
Changhong	*Citrus sinensis*
Kenko	*Citrus reticulata*
Summer gold navel orange	*Citrus sinensis*
Coarse lemon	*Citrus jambhiri*
Zigui tu guang Mandarin	*Citrus sinensis*
Longleaf orange	*Citrus sinensis*
Quzhou early-maturing oranges	*Citrus unshiu*
Nanfeng mandarins	*Citrus reticulata*
Autumn orange	*Citrus reticulata*
Red lemon	*Citrus limonia*
Slippery oranges	*Citrus japonica*

Citrus aurantium (raw)	*Poncirus trifoliata*
Red oranges	*Citrus reticulata*
Yunnan orah mandarin	*Citrus reticulata* × *sinensis*
W. Murcott Citrus	*Citrus reticulata*
Cocktail grapefruit	*Citrus paradisi*
Star ruby grapefruit	*Citrus paradisi*
Quzhou native citrus	*Citrus reticulata*
Citrus aurantium	*Poncirus trifoliata* × *Citrus maxima*
Marshua grapefruit	*Citrus paradisi*

Table_S2 Sensory evaluation results of different varieties of citrus dark tea

Tea samples	Soup color (10 points)	Aroma (30 points)		Taste (50 points)					Score
		Aroma	Coordination	Sour	Sweet	Bitter	Astringent	Coordinate	
Red dark willow navel orange	Red, bright 8.9	Tea fragrance 14.3	Moderately coordinate 8.3	Slightly sour 7	Sweet 8	Not bitter 8	Not astringent 8	Moderately coordinate, slightly sour 7	69.5
Qingjia navel orange	Red, bright 9	Faint fruity aroma, moderately pleasant 14	Moderately coordinate 8	Slightly sour 7	Sweeter 7	Not bitter 8	Not astringent 8	Slightly sour, smooth, more coordinate 8	69
Quzhou medium-ripe oranges	Light red, bright 8.9	Moderately pleasant 15	More coordinated 9	Slightly sour 6.5	Sweeter 7	Not bitter 8	Slightly astringent7	Moderately coordinate 6.5	67.9
Peach leaf orange	Light red, bright 9	Fruity aroma is still 15	Moderately coordinate8.8	Sour 5.7	Sweeter 7	Not bitter 7.7	Slightly astringent 7.7	Moderately mellow, coordinate 7	67.9

Fukumoto navel orange	Deep red, moderately bright 8.9	Moderately pleasant 14.5	Moderately coordinate8.5	Slightly sour 5.5	Sweeter 7	Not bitter 8	Not astringent 8	Slightly sour 7	67.4
Golden orange	Deep red, bright 9	Moderately pleasant 15	More coordinated9	Slightly sour 5	Sweeter 7	Not bitter 8	Slightly astringent7	Moderately coordinate 7	67
Newhall	Deep red, moderately bright 9	Fruity higher 14.5	Moderately coordinate8.5	Slightly sour 6	Sweeter 7	Not bitter 8	Slightly astringent7	Moderately coordinate 7	67
National Day No. 1 Wenzhou mandarin	Deep red, bright 8.8	Fruity 15	Moderately coordinate 8.8	Slightly sour 5	Sweeter 7	Not bitter 8	Not astringent 8	Slightly sour 6	66.6
Klimantin red oranges	Red, bright 9	Weak 14.5	Moderately coordinate 9	Slightly sour 6	Sweeter 7	Not bitter 8	Slightly astringent 6	Moderately coordinate 7	66.5
Early Golden Sweet Orange	Light red, moderately bright 14.3	Fruity aroma, slightly stimulating 14.2	Moderately coordinate 8.3	Slightly sour 5.5	Sweeter 6	Bitter 5.5	Slightly astringent 6	Slightly sour 6.5	66.3
Amber sweet orange	Light red, moderately bright 8.8	Fruity, slightly muted 14	Moderately coordinate 8	Slightly sour 5.5	Sweeter 7	Not bitter 8	Slightly astringent7	Slightly sour 7	65.3
Trifoliate orange	Red, Moderately Bright, with oil beads 8.8	The peach fragrance is still high 16	More coordinated9	Slightly sour 5.5	Sweeter 6	Slightly bitter 7	Slightly astringent 6	Fruity and prominent, slightly thinner 6.5	64.8
Qu tangerine	Red, bright 9	Fruity 14.3	Moderately coordinate8.5	Slightly sour 6	Sweeter 7	Slightly bitter 7	Slightly astringent7	Fishy smell, under coordinate6	64.8
Lime	Red, bright 9	Fruity 15	More coordinated9	Slightly sour 5	Sweeter 7	Slightly bitter 7	Slightly astringent7	Sour 5.5	64.5
Huangyan is local early	Red, moderately bright 8.9	Weak, still pleasant 14.5	Moderately coordinate 7	Slightly sour 6	Sweeter 7	Slightly bitter 7	Slightly astringent 7	Moderately coordinate, slightly sour 7	64.4

Inaba Wenzhou mandarin	Deep red, moderately bright 8.9	Fruity higher 15.2	More coordinated 9.2	Slightly sour 5	Sweeter 7	Slightly bitter 7	Slightly astringent 6	There is a numb mouth feeling of 5.5	63.8
72-1 copper water sweet orange	Light red, moderately bright 8.9	Fruity (sweet) is still high and pleasant 15	Moderately coordinate8.5	Slightly sour 5	Sweeter 6	Slightly bitter 7	Slightly astringent7	Slightly sour, slightly thinner 6	63.4
Kiyomi	Light red, bright 8.8	Moderately pleasant 14.6	Moderately coordinate 8.8	Slightly sour 5	Sweeter 6	Slightly bitter 6.8	Slightly astringent 6.8	Slightly sour 6.3	63.1
Taroko	Red, bright 9	Fruity weak 14	Moderately coordinate 8.5	Slightly sour 5	Sweeter 6	Slightly bitter 7	Slightly astringent7	Slightly sour 6.5	63
Red-fleshed Peach leaf orange	Red, bright 9	Moderately pleasant 14.9	More coordinated 9	Slightly sour 6	Sweeter 6	Slightly bitter 6	Slightly astringent 6	Slightly sour 6	62.9
Big red sweet orange	Light red, bright 8.9	Fruity (sweet), still pleasant 14.3	More coordinated 7.7	Slightly sour 5.9	Sweeter 6	Slightly bitter 7	Slightly astringent 7	Slightly sour 6	62.8
Minaxia orange	Red, bright 9	Fruity and pleasant 14.8	More coordinated 8.8	Slightly sour 5	Sweeter 6	Slightly bitter 6.5	Slightly astringent 6.5	Moderately coordinated, slightly sour 6	62.6
Quzhou mandarin orange	Red, slightly darker 8.8	Slightly greenish 14	Moderately Coordinate 8	Slightly sour 6.5	Sweeter 6	Slightly bitter 6	Slightly astringent7	Moderately Coordinate 6	62.3
Ehime	Red, bright 8.9	Fruity (lemon) high 15	More coordinated 9	Slightly sour 5	Sweeter 6	Slightly bitter 6	Slightly astringent 6	Slightly sour 6.2	62.1
Ortanic	Red, bright 9	Weak, fruity 14.5	Slightly different, moderately coordinated 8.5	Slightly sour 5	Sweeter 7	Slightly bitter 6	Slightly astringent 6	Slightly sour 6	62

Seedless ponkan	Red, bright 9	Tall, still pleasant15	More coordinated 9	Slightly sour 5	Sweeter 6	Slightly bitter 6	Slightly astringent 6	Slightly sour, moderately coordinated 6	62
Hamorim sweet orange	Red, bright 9	Fruity, slightly dull and dull14	Slightly coordinated 6.5	Slightly sour 6	Sweeter 6	Slightly bitter 7	Slightly astringent7	Moderately coordinated, slightly sour 6.5	62
Xinnv ponkan	Deep red, bright 9	Fruity (sweet) is still high, more pleasant 14.8	Moderately coordinate 8.5	Slightly sour 6	Sweeter 6	Bitter 5.8	Slightly astringent 6	Moderately coordinated, slightly sour 5.9	62
Maogu mandarin	Red, bright 9	The fruit (sweet) aroma is more pleasant 14.9	Moderately coordinate 8.9	Slightly sour 5.5	Sweeter 6	Bitter 5.8	Slightly astringent 6	Moderately coordinated 5.9	62
Yungui orange	Light red, bright 8.9	Fruity aroma is weak, still pleasant 14.2	Moderately coordinate 8.6	Slightly sour 6	Sweeter 6	Slightly bitter 6	Slightly astringent 6	Slightly sour, moderately coordinated 6.2	61.9
Sunshine orange	Light red, bright 8.9	Moderately pleasant 14	Moderately coordinate 7	Slightly sour 5.5	Sweeter 6	Slightly bitter 7	Slightly astringent7	Slightly sour, moderately coordinated 6.5	61.9
Voling summer orange	Red, bright 9	Fruity high 15	Moderately coordinate 8.9	Slightly sour 5	Sweeter 6	Slightly bitter 6	Slightly astringent 6	Under coordinated, slightly sour 5.5	61.4
Yuanhong	Light red, bright 8.9	Fruity weak 14	Moderately coordinate 8	Slightly sour 6	Sweeter 6	Slightly bitter 6	Slightly astringent 6	Moderately coordinate 6	60.9
Orange grapefruit	Light red, moderately bright 8.8	Fruity high, slightly stimulating 14.3	Moderately coordinate 8.3	Slightly sour 5.5	Sweeter 6	Slightly bitter 6	Slightly astringent 6	Moderately coordinated, slightly sour 5.8	60.7
Mandarin oranges	Deep red, moderately bright 9	The fruity aroma is still high, and it is still pleasant 14.3	More coordinated 7.2	Slightly sour 6.3	Sweeter 6	Bitter 5.8	Slightly astringent 6	Slightly sour, slightly bitter 5.8	60.4
Citrus aurantium	Red, bright 9	Slightly fishy 9	Under-coordinate 4	Slightly sour 7	Sweet 8	Not bitter 8	Not astringent 8	Sweet alcohol 7.3	60.3

Red-fleshed navel orange	Orange-red, moderately bright 8.9	The fruity aroma is still high, and it is still pleasant 14.8	Moderately Coordinate 8.5	Slightly sour 5	Sweeter 6	Slightly bitter 6	Astringent 5.5	Under coordinated, slightly sour 5.5	60.2
Taiwan ponkan	Deep red, bright 9	Fruity higher 15.1	More coordinated 9	Slightly sour 5	Sweeter 6	Slightly bitter 5	Astringent 5	Still coordinate, slightly sour, slightly bitter6	60.1
Brown orange	Red, bright 9	Fruity, still pleasant 14.5	Moderately coordinate 8.5	Slightly sour 5	Sweeter 6	Slightly bitter 6	Astringent 5.5	Slightly sour 5.5	60
Huanong local early	Deep red, moderately bright 8.9	Fruity 14.5	Moderately coordinate8.5	Slightly sour 5	Sweeter 6	Slightly bitter 7	Astringent 5	Slightly sour 5	59.9
Ignorant fire	Orange-red, bright 8.9	Fruity weak 14	Moderately Coordinate 8.2	Slightly sour 5	Sweeter 6	Bitter 5.7	Slightly astringent 6	Slightly sour 6	59.8
Red crisp navel orange	Red, brighter 8.8	The fruity aroma is weak, but it is still pleasant14	Moderately Coordinate 8	Slightly sour 6	Slightly sweeter 5	Slightly bitter 6	Slightly astringent 6	Slightly sour, moderately Coordinated 6	59.8
Large-fruited summer orange	Light red, bright 9	Fruity, still pleasant 13.8	Moderately coordinate 7.9	Slightly sour 5	Sweeter 6	Slightly bitter 6	Slightly astringent 6	It has a fishy smell, slightly sour6	59.7
Gannan early	Red, bright 9	Still high, still pleasant 14.8	Moderately coordinate8.8	Slightly sour 5.5	Sweeter 6	Bitter 5	Astringent 5	Slightly sour, bitter, and uncoordinated5	59.1
Grapefruit	Red, bright 9	Fruity 15	Moderately coordinate 9	Slightly sour 7	Sweeter 6	Bitter 4	Astringent 5	Bitter, under coordinated4	59
Navelina navel orange	Red, bright 9	Fruity and high 15	More coordinated 9	Slightly sour 6	Slightly sweeter 5	Bitter 5	Astringent 5	Slightly bitter, slightly sour, under coordinated 5	59
Qingjiang mandarin	Red, bright 9	The fruity aroma is still high, and it is still pleasant 15	Moderately Coordinate8.9	Slightly sour 5	Sweeter 6	Bitter 5	Astringent 5	Under Coordinate, slightly sour5	58.9

Thin-peel sweet orange	Light red, bright 8.9	The fruity aroma is still high, and it is still pleasant 14.8	Moderately coordinate 7	Sour 4.5	Sweeter 6	Slightly bitter 6	Slightly astringent 6	More sour 5.5	58.7
American granulated sugar oranges	Light red, bright 8.9	Moderately pleasant 14.3	Moderately coordinate8.4	Slightly sour 6	Sweeter 6	Bitter 5	Astringent 5	Slightly sour, bitter 5	58.6
Lun night	Red, dark 8.8	Fruity and high 15	More coordinated 9	Slightly sour 5	Slightly sweeter 5	Slightly bitter 6	Astringent 5	Slightly sour, undercoordinated 4.5	58.3
Xinhui mandarin	Red, bright 9	Moderately pleasant 13.8	Moderately coordinate 6.3	Slightly sour 5.5	Sweeter 6	Bitter 5.5	Slightly astringent 6	Moderately coordinated, slightly sour 6	58.1
September red navel orange	Red, moderately bright 8.9	Weak 14.2	Moderately coordinate 8.1	Slightly sour 5.7	Slightly sweeter 5	Bitter 5.5	Astringent 5	More sour, less coordinated 5.3	57.7
Yichang orange	Red, bright, with oil beads 8.9	Slightly fruity 14.2	Moderately Coordinate8.5	Slightly sour 5	Slightly sweeter 5	Slightly bitter 6	Astringent 5	Moderately Coordinated, more sour and astringent 5	57.6
Changhong	Orange-red, bright 8.9	Higher, still pleasant 14	Moderately Coordinate 8.1	Slightly sour 5.5	Slightly sweeter 5	Slightly bitter 6	Astringent 5	Slightly sour and astringent, undercoordinated 5	57.5
Kenko	Light red, bright 8.9	Fruity and slightly pungent13	Moderately coordinate 7	Slightly sour 5.5	Sweeter 6	Slightly bitter 6	Slightly astringent 6	The orange flavor is pronounced and uncoordinated5	57.4
Summer gold navel orange	Red, slightly darker 8.8	The fruity aroma is more stimulating 14.3	More coordinated 8.5	Slightly sour 5	Slightly sweeter 5	Bitter 5	Astringent 5.5	Under Coordinate 5	57.1
Coarse lemon	Red, slightly darker 8.9	The fruity aroma is still high and pleasant 15	More coordinated 9	Slightly sour 5	Slightly sweeter 5	Bitter 4.2	Astringent 5	Under Coordinate, bitter 4.5	56.6

Zigui tu guang Mandarin	Light red, bright 8.9	Fruity and pleasant 14	Moderately coordinate 7	Slightly sour 5	Sweeter 6	Bitter 5.3	Astringent 5.3	Bitter, slightly sour 4.9	56.4
Longleaf orange	Red, bright 9	Moderately pleasant 14.1	Moderately coordinate 8.1	Slightly sour 5.5	Sweeter 6	Bitter 5	Astringent 4	More sour, less coordinated 4.5	56.2
Quzhou early-maturing oranges	Red, bright 9	Fruity weak 14	Moderately coordinate 8	Slightly sour 5	Sweeter 6	Bitter 4	Slightly astringent 6	Slightly sour and bitter 4	56
Nanfeng mandarins	Red, bright 9	Slightly muted, still strong 13	Moderately coordinate 6	Slightly sour 5.5	Sweeter 6	Slightly bitter 6	Astringent 5	Slightly sour, astringent 5.5	56
Autumn orange	Red, bright 9	Dull and unpleasant 13	Under coordinate 5	Slightly sour 5.5	Sweeter 6	Slightly bitter 6	Astringent 5.7	Slightly sour 5.5	55.7
Red lemon	Red, bright 9	Moderately pleasant 14.8	Moderately coordinate 8.8	Sour 4	Slightly sweeter 5	Bitter 5	Astringent 5	Acid, incongruous 4	55.6
Slippery oranges	Red, slightly darker 8.8	Fruity 14	Moderately Coordinate 8	Slightly sour 5	Slightly sweeter 5	Bitter 5	Astringent 5	Lack of Coordinate 4.5	55.3
Citrus aurantium (raw)	Red, bright, with oil beads 9	Moderately pleasant 15	More coordinated 9	Slightly sour 5	Slightly sweeter 5	Bitter 4	Astringent4	Sour, bitter 4	55
Red oranges	Red, bright 9	Fruity and pleasant, slightly dull and dull.14	Moderately coordinate 8	Slightly sour 5	Slightly sweeter 5	Bitter 4	Astringent 5	Under coordinate, bitter 4.5	54.5
Yunnan orah mandarin	Red, bright 9	Slightly muted 13.3	Moderately coordinate 6.7	Slightly sour 5	Sweeter 6	Bitter 5	Astringent 5	More sour, less coordinated 4.5	54.5
W. Murcott Citrus	Deep red, slightly darker 8.8	Unpleasant 13	Moderately Coordinate 7	Sour 4.5	Slightly sweeter 5	Bitter 5	Astringent 4.5	Under coordinated, more sour 4.8	52.6
Cocktail grapefruit	Red, bright 9	The fruity aroma is still high, and it is still pleasant 15	More coordinated 9	Slightly sour 5	Slightly sweeter 4	Bitter 4	Astringent 3	Incongruous, sour, bitter 3	52

Star ruby grapefruit	Red, slightly darker 8.8	Slightly lower, still pleasant 14.5	Moderately Coordinate 8.5	Slightly sour 5	Slightly sweeter 4	Bitter 4	Astringent 3	Incongruous, sour, bitter 3	50.8
Quzhou native citrus	Red, bright 9	Fishy, fragrant, unpleasant9	Under-coordinate 4	Slightly sour 5.3	Sweeter 6	Bitter 5.5	Astringent 5.5	The fragrance is rich, similar to Xinhui mandarin 5.5	49.8
Citrus aurantium	Red, bright 9	Slightly grassy 14	Moderately coordinate 8	Sour 4	Slightly sweeter 4	Bitter 3	Astringent4	Incongruous, sour, bitter, grapefruit3	49
Marshua grapefruit	Light red, bright 8.8	Slightly muted 14	Moderately coordinate 8	Slightly sour 5	Slightly sweeter 4	Bitter 3	Astringent3	Incongruous, sour, bitter 3	48.8

Table_S3 Sensory quality of Peach leaf orange dark tea at different harvest times

Harvest time	Soup color	Aroma		Taste					Score
	Comments	Comments	Coordinate	Sour	Sweet	Bitter	Astringent	Coordinate	
August	Red bright 9±0.1	The fruity aroma is still high 15±0.1	Moderately coordinated 9±0	Sour 5.7±0.6	Sweeter 6±0 b	Slightly bitter 6±0 b	Astringent 5.7±0.2 b	Moderately coordinated 5.8±0.4 b	62.1±0.8 c
September	Red bright 9±0.1	The fruity aroma is still high 14.9±0.1	Moderately coordinated 8.9±0.1	Slightly sour 6±0.3	Sweeter 7±0 a	Slightly bitter 7.2±0.4 a	Slightly astringent 7.2±0.4 a	Coordinated 7±0 a	67.2±0.6 a
October	Red bright 8.9±0.1	Moderately pleasant 14.9±0.1	Moderately coordinated 8.9±0.1	Sour 5.9±0.2	Sweeter 6.1±0.2 b	Slightly bitter 6.1±0.9 b	Slightly astringent 6.2±0.8 a	Moderately coordinated 6.1±0.7 b	63.2±1.8 bc
November	Red bright 9±0.1	Moderately pleasant 15±0.1	Moderately coordinated 9±0	Slightly sour 6.3±0.6	Sweeter 6.1±0.17 b	Slightly bitter 6.2±0.7 b	Slightly astringent 6.3±0.6 a	Moderately coordinated 6.3±0.4 b	64.2±1.2 b
December	Red bright 9±0.1	Moderately pleasant 14.9±0.2	Moderately coordinated 8.9±0.1	Slightly sour 6.1±0.6	Sweeter 6±0 b	Bitter 5.8±0.3 b	Slightly astringent 6±0 a	Moderately coordinated 6.0±0.6 b	62.8±1.2 c

Note: The LSD method was used for multiple comparisons, and different letters in the same column (row) indicated significant differences in the level of $p < 0.05$.

Table_S4 Analysis of volatile components in orange tea with different maturity levels (μg/g)

RT	RI	Compound name	August	September	October	November	December
		**Alcohols**					
9.26	1070	1-Octanol	35.22±4.56a	37.62±1.83a	27.17±1.22b	25.24±0.65b	20.89±0.82a
9.84	1086	Linalool oxide II	6.58±0.51b	6.55±0.17b	4.68±0.44c	5.35±0.51c	7.55±0.32a
10.45	1102	Linalool	126.29±9.56a	96.4±1.31b	93.7±5.1b	79.16±2.44c	98.97±5.43a
10.83	1111	Phenethyl alcohol	14.68±1.67a	15.35±0.66a	13.88±1.31c	12.05±1.03b	13.15±1.06a
11.29	1122	(E)-para-2, 8-1-menthadienol	26.85±1.45b	22.92±0.78c	29.64±0.64a	17.16±0.99e	17.17±0.79d
12.56	1151	Isopulegol	1.78±0.2a	1.28±0.02c	1.24±0.09c	1.27±0.09c	2.15±0.11b
13.67	1173	1-Nonanol	9.7±1.04a	7.1±0.13cd	7.22±0.52bc	6.15±0.34d	6.01±0.49 ab
13.84	1175	Linalool oxide (pyranoid)	3.6±0.27b	5.78±0.16a	2.67±0.12c	3.79±0.46b	3.72±0.37b
14.11	1181	Terpinen-4-ol	35.64±1.6a	23.61±0.45c	21.15±1.07d	15.01±0.31e	21.67±4.82b
14.45	1187	Isocarveol	41.86±3.32b	38.46±2.84b	59±5.57a	23.5±2.53c	23.36±0.62c
14.88	1195	L-α-Terpineol	62.49±4.12a	47.29±1.41b	45.14±3.73b	34.78±2.58c	33.43±4.91b
15.07	1198	Isopiperitenol	19.85±0.68b	19.99±1.31b	23.59±2.06a	16.44±0.44c	17.17±1.31b
15.26	1202	1, 6-Dihydrocarveol	-	2.07±0.2	-	-	-
16.04	1218	trans-Carveol	62.09±5.91a	54.95±0.92b	63.73±2.44a	45.61±2.57c	44.66±1.52b
16.35	1224	Nerol	26.44±2.81a	21.2±0.37b	15.83±0.7c	7.45±0.39e	11.22±0.38d
16.76	1232	cis-Carveol	25.2±3.08a	21.86±0.31ab	20.54±1.28b	18.87±1.79b	21.09±1.34a
17.85	1251	Geraniol	19.14±2.47a	13.05±0.22b	9.81±0.2c	7.25±0.23d	11.96±0.26b
17.98	1254	2-Methoxybenzyl alcohol	3.29±0.06	3.41±0.47	3.68±0.3	3.49±0.34	2.77±0.26
20.06	1288	p-Mentha-1 (7), 8 (10)-dien-9-ol	21.7±2.6b	13.3±0.5d	23.27±0.35b	15.96±0.97c	26.0±0.9a
31.34	1501	Cubebol	1.42±0.11a	0.81±0.08bc	0.91±0.12b	0.7±0.06c	0.69±0.05bc
32.99	1537	Elemol	13.22±1.18a	6.14±0.47c	5.67±0.75c	3.68±0.33d	6.47±0.17b
33.77	1554	(E)-Nerolidol	4.82±0.53a	2.6±0.32b	2.46±0.29b	2.29±0.32b	2.52±0.24b
34.21	1563	Spathulenol	16.18±0.85a	7.81±0.45cd	8.59±0.38c	7.27±0.75d	8.3±0.37b
35.57	1590	Cedrol	3.5±0.12a	1.65±0.1d	1.95±0.35c	1.47±0.04e	2.48±0.02b
37.14	1638	Cubenol	3.05±0.34a	1.65±0.21b	2.67±0.31a	1.77±0.24b	2.26±0.13a
39.02	1704	Farnesol	-	-	-	-	2.68±0.01

44.55	2106	Phytol	1.49±0.14a	0.42±0bc	0.52±0.09b	0.27±0.01c	0.35±0.05bc
		**Aldehydes and ketones**					
4.18	856	(E)-2-Hexenal	0.78±0.05	0.88±0.05ab	0.76±0.02b	0.82±0.02ab	0.71±0.05ab
4.93	901	Heptanal	1.67±0.04	-	-	-	-
6.02	955	(E)-2-Heptenal	0.88±0.25c	1.84±0.15a	1.5±0.12b	0.56±0.06c	0.35±0.01c
6.17	961	Benzaldehyde	1.74±0.22c	2.69±0.1a	2.21±0.15b	2.3±0.08b	1.23±0.06d
7.19	1003	Octanal	14.67±1.05c	27.06±1.15a	28.48±4.08a	21.96±1.04b	11.94±0.48c
7.38	1010	(E,E)-2, 4-Heptadienal	9.99±0.84ab	10.96±0.13a	9.79±0.54ab	9.29±0.88b	8.83±0.44a
8.39	1044	Benzeneacetaldehyde	1.21±0.02b	1.85±0.29a	1.31±0.11b	1.32±0.14b	0.94±0.06b
10.56	1105	Nonanal	12.25±0.91b	11.76±0.74b	14.14±1.47a	11.22±0.24b	10.11±0.75b
11.5	1127	α-Campholenal	-	-	-	-	0.56±0.06
12.71	1154	Citronellal	5.14±0.49a	3.58±0.35b	2.88±0.13c	1.71±0.1d	3.31±0.45b
12.9	1157	Sabina ketone	-	-	-	-	0.94±0.03
13.08	1161	(E)-2-Nonenal	2.91±0.39a	2.49±0.18ab	1.7±0.18c	2.13±0.17bc	2.5±0.29ab
15.43	1206	Decanal	40.2±1.56b	38.97±0.78b	48.69±1.79a	36.87±1.39b	33.82±2.87b
15.65	1210	Verbenone	5.55±0.5b	5.92±0.23ab	6.64±0.47a	4.19±0.42c	4.55±0.37b
17.1	1238	β-Citral	21.23±1.5a	17.32±1.14b	14.04±0.49c	11.3±0.8d	13.6±0.58b
17.43	1242	Carvone	50.87±4.46b	49.41±0.75b	57.84±1.5a	53.41±3.85ab	55.9±4.9ab
18.53	1263	trans-2-Decenal	5.17±0.53ab	5.81±0.44a	4.55±0.25b	4.95±0.45b	4.24±0.25b
18.86	1269	Citral	39.5±5a	36.71±0.57ab	32.63±0.64b	27.8±0.81c	25.07±1.85b
19.16	1274	Perillaaldehyde	36.97±4.87a	36.16±1.72a	38.22±4.31a	27.94±1.44b	26.02±2.77a
20.28	1291	2-Undecanone	2.5±0.28b	3.29±0.26a	2.77±0.11b	2.4±0.13b	2.21±0.14b
21.11	1306	Undecanal	8.24±1.18a	6.74±0.82ab	7.89±0.25a	6±0.45b	5.38±0.74b
23.74	1358	Nerol acetate	1.17±0.16a	0.81±0.06b	0.61±0.06c	0.34±0.09e	0.33±0.05d
24	1363	2-Undecenal	-	-	6.17±0.15	4.63±0.19	4.88±0.76
26.31	1405	Dodecanal	11.65±1.42b	10.45±0.91d	16.02±0.02a	11.23±0.92cd	10.41±0.23bc
26.7	1413	α-Ionone	7.41±0.37a	7.17±0.12a	7.2±0.93a	5.86±0.33b	4.57±0.41b
28.01	1439	Neryl acetone	19.57±1.04ab	20.05±1.25a	19.39±1.85ab	17.39±0.73bc	13.79±0.93c
29.48	1467	trans-β-Ionone	13.61±0.44a	10.17±0.44bc	10.73±0.75b	8.96±0.42 cd	7.96±0.21e
29.64	1470	β-Ionone epoxide	6.53±0.45a	5.05±0.26c	5.68±0.47bc	5.04±0.3c	5.28±0.09ab
34.72	1573	(E)-ψ-Ionone	5.57±0.52	3.26±0.37	-	-	-

38.51	1685	β-Sinensal	10.35±1.27a	4.46±0.41b	2.89±0.27c	1.81±0.32d	3.58±0.31b
40.58	1790	Nootkanone	-	-	-	-	2.32±0.14
41.28	1837	Hexahydrofarnesylacetone	9.81±1.06a	6.75±0.11bc	7.58±0.75b	5.79±0.27c	6.4±0.14b
42.22	1904	Farnesyl acetone	0.84±0.26b	0.67±0.03b	0.88±0.17b	0.81±0.07b	1.13±0.05a
42.43	1921	Hexadecanoic acid, methyl ester	3.21±0.39a	1.5±0.01c	2.02±0.27b	1.54±0.23c	2.95±0.07a
42.76	1950	Dibutyl phthalate	1.11±0.11a	0.21±0.01c	0.31±0.01c	0.26±0.01c	0.76±0.1b
		**Alkenes of olefins**					
5.6	935	α-Pinene	4.79±0.55a	3.58±0.44b	2.74±0.35cd	2.44±0.11d	2.88±0.25bc
6.47	974	Sabinene	6.5±0.82	-	-	-	-
6.83	989	β-Myrcene	9.78±0.92a	9.53±1.37a	7.89±0.07b	8.7±0.7ab	3.55±0.02c
7.61	1018	α-Terpinene	2.61±0.13a	1.6±0.21b	1.38±0.08bc	1.18±0.09c	1.02±0.11c
7.83	1026	o-Cymene	9.48±0.72b	11.18±0.35a	7.94±0.88c	6.07±0.17d	5.16±0.15e
8.05	1033	Limonene	200.85±7.48a	193.62±30.62a	188.78±9.88a	186.12±13.18a	122.23±10.19b
8.87	1059	γ-Terpinene	9.72±0.63a	8.53±0.88b	7.73±0.65bc	6.75±0.33cd	5.37±0.43d
9.97	1090	p-Cymenene	2.03±0.26c	2.83±0.17b	3.69±0.13a	1.88±0.03c	0.77±0.05d
10.88	1113	1, 3, 8-p-Menthatriene	-	-	5.61±0.72	-	-
11.67	1131	4-Acetyl-1-methylcyclohexene	1.96±0.07b	1.92±0.18b	1.93±0.01b	2.48±0.31a	2.47±0.12a
24.38	1370	(-)-α-Pinene	20.59±0.46a	14.62±0.69d	17.72±1.01b	15.58±0.9cd	15.31±1.68cd
25	1381	β-cubebene	4.9±0.21 a	2.33±0.09c	3.05±0.13b	2.05±0.23c	2.09±0.33c
25.1	1383	β-Elemene	9.55±0.38 a	6.28±0.37b	6.44±0.03b	4.02±0.42c	4.07±0.35c
26.47	1408	β-Longipinene	5.93±0.28a	3.17±0.34b	2.98±0.21b	2.98±0.41b	5.26±0.59a
26.96	1418	Cedrene	11.25±1.08bc	9.66±0.45c	12.41±0.99ab	11.19±0.49bc	12.78±0.97a
28.15	1442	Humulene	2.73±0.18a	1.23±0.1b	1.23±0b	0.97±0.13c	1.05±0.08b
28.29	1445	cis-β-Farnesene	4.92±0.48c	2.2±0.26b	2.22±0.12b	0.82±0.11c	0.75±0.17c
29.04	1459	Alloaromadendrene	-	-	2.25±0.13	1.29±0.09	1.64±0.24
29.23	1462	γ-Muurolene	5.79±0.52a	2.01±0.26c	4.04±0.43b	3.35±0.32b	4.9±0.53a
29.88	1474	β-Selinene	2.7±0.32a	1.73±0.16c	2.38±0.11b	1.75±0.14c	2.39±0.18b
30.11	1479	Valencene	4.63±0.35c	3.31±0.17c	3.91±0.21c	8.55±0.89b	16.73±1.92a
30.51	1486	α-Muurolene	6.26±0.45a	3.56±0.25c	4.58±0.34b	3.72±0.36c	3.36±0.14c
31.54	1505	β-cadinene	27.24±1.89a	9.28±0.23d	10.44±0.98b	10.04±0.05b	9.68±0.86c
32.17	1519	Cubenene	4.24±0.11a	2.03±0.16c	3.06±0.11b	1.71±0.1d	1.83±0.24d

32.55	1528	α-Calacorene	6.51±0.47a	3.98±0.11bc	4.61±0.16b	3.68±0.51c	3.43±0.09c
		**Acid &Asters**					
9.45	1075	Heptanoic acid	1.68±0.17a	1.78±0.09a	1.12±0.05b	0.94±0.05b	0.79±0.02c
19.36	1277	Nonanoic acid	4.57±0.12b	5.29±0.43a	5.09±0a	4.28±0.44b	2.13±0.24c
21.77	1319	trans-Geranic acid methyl ester	7.28±0.4	6.56±0.06	4.71±0.45	3.7±0.19	3.8±0.32
24.02	1363	Butyl carbitol acetate	7.45±0.64	6.05±0.14	-	-	-
24.74	1376	Geranyl acetate	-	-	-	-	1.96±0.11
24.79	1377	Decanoic acid	15.47±0.65	-	-	-	-
25.83	1396	N-Methylmethylanthranilate	11.38±0.01b	15.13±0.36a	8.16±0.22c	2.36±0.27e	2.5±0.34d
31.72	1509	Dihydroactinidiolide	25.06±1.35	22.16±0.54	22.75±2.68	22.27±2.33	20.13±1.14
42.86	1958	Hexadecanoic acid	13.01±0.86a	4.07±0.31c	6.44±0.69b	4.16±0.22c	10.76±1.36a
		**Others**					
8.5	1047	1-Ethyl-2-formylpyrrole	4.1±0.16a	4.27±0.18a	2.68±0.15c	2.73±0.38c	2.89±0.27b
12.87	1157	β-Pineneoxide	5.19±0.65	3.16±0.21	5.71±0.19	-	-
19.67	1282	3, 4-Diethylphenol	9.6±0.82b	12.32±0.68a	7.45±0.87c	4.15±0.4d	4.11±0.85d
20.15	1289	Thymol	5.81±0.67	-	-	-	-
20.59	1296	Carvacrol	21.43±1.3b	24.93±0.84a	14.49±0.79c	9.44±0.69d	9.43±1.17d
20.76	1299	Dipentene diepoxide	3.2±0.25b	3.93±0.19a	1.93±0c	1.48±0.13d	1.39±0.06d
20.86	1300	1-Methylnaphthalene	0.93±0.11b	1.08±0.08b	0.97±0.09b	1.45±0.11a	1.23±0.14a
21.01	1304	1, 2, 3-Trimethoxybenzene	12.69±0.99a	12.85±1.31a	12.21±1.21a	11.94±1.28ab	11.15±1.32ab
23.18	1347	Eugenol	-	-	-	-	4.89±0.36
24.17	1366	1, 2, 4-Trimethoxybenzene	11.82±0.58d	13.4±0.64b	14.19±0.75a	13.7±0.54b	12.79±1.39c
30.81	1491	Butylated hydroxytoluene	-	-	1.79±0.11	1.12±0.11	-
32.23	1521	1, 2, 3, 4-Tetramethoxybenzene	2.76±0.09b	2.26±0.21c	3.04±0.2a	2.57±0.23b	2.17±0.05c
34.4	1567	Caryophyllene oxide	13.48±0.75a	11.51±0.6b	11.35±0.46b	7.54±0.69c	12.2±1.38b
35.66	1592	Humulene epoxide II	8.81±0.62a	5.5±0.09b	6.37±0.51b	6.57±0.59b	5.14±0.76c
40.09	1764	Phenanthrene	1.96±0.14	0.31±0.02	-	-	-

Note: The LSD method was used for multiple comparisons, and different letters in the same column (row) indicated significant differences in the level of $p < 0.05$. The measured exact mass for all identified compounds showed a deviation of less than 500 ppm from their theoretical values. All compounds were identified with Level 2 confidence (putative annotation) per MSI guidelines, supported by mass spectral library matching and retention index comparison.

Table_S5 Differential aroma component content of orange dark tea at different harvest times (μg/g)

	August	September	October	November	December
Limonene	200.85±7.48	193.62±30.63	188.78±9.88	186.12±13.18	143.75±11.99
trans-Carveol	62.09±5.91	54.95±0.92	63.73±2.45	45.61±2.57	52.52±1.8
Isocarveol	41.86±3.32	38.46±2.84	59±5.58	23.5±2.53	27.48±0.74
Decanal	41.07±2.17	37.51±2.65	48.69±1.8	36.87±1.39	39.77±3.38
Perillaaldehyde	36.97±4.88	36.16±1.72	38.22±4.31	27.94±1.45	36.02±2.33
1-Octanol	35.22±4.56	37.62±1.84	27.17±1.23	25.24±0.66	35.69±1.75
(E)-para-2, 8-1-menthadienol	26.85±1.46	22.92±0.78	29.64±0.65	17.16±0.99	20.19±0.94
p-Mentha-1 (7), 8 (10)-dien-9-ol	21.71±2.6	13.34±0.52	23.27±0.36	15.96±0.97	25.99±0.92
Carvacrol	21.43±1.31	24.93±0.85	14.49±0.79	9.44±0.69	13.17±0.63
Isopiperitenol	19.85±0.68	19.99±1.31	23.59±2.07	16.44±0.44	20.19±1.54
Phenethyl alcohol	14.68±1.68	15.35±0.66	8.07±0.7	12.05±1.03	15.47±1.25
Dodecanal	14.25±1.37	10.19±0.95	16.02±0.03	11.23±0.93	12.8±0.98
Caryophyllene oxide	13.48±0.76	11.51±0.6	11.35±0.47	7.54±0.7	14.35±1.62
Nonanal	12.25±0.92	11.76±0.75	14.14±1.48	11.22±0.25	11.89±0.89
N-Methylmethylanthranilate	11.38±0.01	15.13±0.36	8.16±0.23	2.36±0.27	3.26±0.45
β-Myrcene	9.78±0.93	9.53±1.37	7.44±0.79	8.99±0.87	4.18±0.03
3, 4-Diethylphenol	9.6±0.82	12.32±0.69	7.45±0.87	3.77±0.31	5.41±0.08
o-Cymene	9.48±0.72	11.18±0.35	7.94±0.88	6.07±0.18	6.65±1.02
Humulene epoxide II	8.81±0.63	5.5±0.1	8.32±0.82	6.57±0.59	6.05±0.9
Linalool oxide II	6.58±0.51	6.55±0.17	4.68±0.44	5.35±0.51	8.88±0.38
(E)-ψ-Ionone	5.57±0.52	3.26±0.37	-	-	-
Verbenone	5.55±0.51	5.92±0.23	6.64±0.47	4.19±0.42	5.35±0.44
trans-2-Decenal	5.17±0.54	5.81±0.44	4.55±0.26	4.95±0.46	4.99±0.29
Valencene	4.63±0.35	3.31±0.18	3.91±0.21	8.55±0.89	19.68±2.26
Nonanoic acid	4.31±0.47	5.29±0.44	5.09±0.01	4.28±0.45	2.51±0.29
1-Ethyl-2-formylpyrrole	4.1±0.16	4.27±0.18	2.68±0.15	2.73±0.39	3.4±0.32

Linalool oxide (pyranoid)	3.6±0.28	6.11±0.59	2.67±0.12	3.79±0.47	4.37±0.44
Cedrol	2.91±0.4	2.49±0.18	1.7±0.19	2.13±0.17	2.95±0.35
(E)-2-Nonenal	2.67±0.27	3.93±0.19	1.93±0	1.48±0.14	1.94±0.21
Dipentene diepoxide	2.5±0.28	3.29±0.26	2.77±0.11	2.4±0.13	2.6±0.18
2-Undecanone	2.03±0.27	2.83±0.18	3.69±0.14	1.88±0.03	0.91±0.06
p-Cymenene	1.68±0.17	1.78±0.09	1.12±0.06	0.94±0.06	2.97±0.19
Heptanoic acid	1.29±0.07	6.56±0.07	4.71±0.46	3.7±0.2	4.47±0.38
trans-Geranic acid methyl ester	1.17±0.12	1.08±0.09	0.97±0.09	1.45±0.12	1.45±0.17
1-Methylnaphthalene	0.58±0.07	1.84±0.15	1.5±0.12	0.56±0.07	0.41±0.02
(E)-2-Heptenal	-	1.6±0.21	1.38±0.08	1.18±0.1	1.2±0.14
